# Developmental Stage‐Specific Responses to Extreme Climatic Events and Environmental Variability in Great Tit Nestlings

**DOI:** 10.1111/gcb.70794

**Published:** 2026-03-11

**Authors:** Devi Satarkar, David López‐Idiáquez, Irem Sepil, Ben C. Sheldon

**Affiliations:** ^1^ Edward Grey Institute of Field Ornithology, Department of Biology University of Oxford Oxford UK

**Keywords:** climate change, developmental effects, early‐life conditions, extreme climatic events, fledging mass, life‐history traits, long‐term studies, post‐fledging survival, wild birds

## Abstract

Climate change poses a pervasive threat to many aspects of natural systems, and while impacts of changes in average conditions have been extensively studied, the effects of increased climate variability and extreme events on natural populations are less understood due to the challenges of studying these rare and unpredictable occurrences. Using 60 years of life‐history data from over 83,000 individuals and historical daily climate records, we show that developmental stages in wild great tits ( *Parus major* ) differ in their sensitivity to extreme climatic events (ECEs). Exposure to extreme cold events during the first week of development is particularly detrimental to fledging mass, while extreme rain events have a stronger negative impact as nestlings grow older and their energetic requirements increase. Synergistic effects of ECEs and average climatic conditions can be particularly severe, exacerbating the challenges faced by these birds. Our findings indicate that combined exposure to extreme heat and heavy rainfall during early development is associated with a predicted reduction in fledging mass by up to 27%. Additionally, birth timing may further modulate these effects, since late‐season broods exposed to frequent hot ECEs during early development are predicted to fledge nestlings up to 4.27 standard deviations (35%) lighter than broods laid earlier in the season. Moreover, phenotypic plasticity has enabled many similar populations to shift towards an overall earlier laying date, which may have increased susceptibility to cold extremes during development. However, our analyses suggest that the benefits of being part of an early‐laid clutch within a season may, to some extent, offset the negative effects of extreme climate on fledging mass and apparent survival. In climate scenarios where ECEs are predicted to increase in frequency, duration and severity, these developmental stage‐specific insights are important for understanding how climate change may be influencing wild avian populations.

## Graphical abstract

Extreme climatic events (ECEs) impact great tit nestlings in a developmental stage‐specific and context‐dependent manner. Using 60 years of data on 83,000+ great tit nestlings from Wytham Woods, UK, we show that cold snaps are detrimental to hatchlings, while older nestlings are more affected by extreme rain and heat. Combined effects of ECEs with ambient weather conditions can worsen outcomes. Our findings indicate that combined exposure to extreme heat and heavy rainfall during early development is associated with a predicted reduction in fledging mass by up to 27%. These insights are important for predicting how wild populations may fare amid the ongoing climate crisis, where ECEs continue to increase in frequency and intensity.

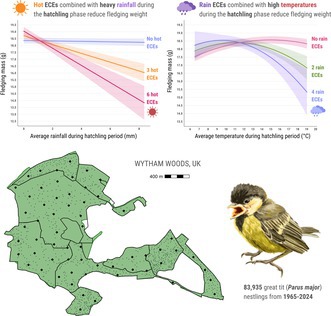

## 1 Introduction

Anthropogenic climate change has warmed the atmosphere at an unprecedented rate in recent decades, posing a profound and multifaceted threat to biodiversity (Urban et al. 2016). Animals are exhibiting diverse responses to changing average climates at the individual and population level, adjusting their phenology, demographic patterns, and spatial distributions (Johnston et al. 2019; Thackeray et al. 2016). For instance, there is well‐documented evidence that plant and animal populations are shifting their ranges poleward or to higher altitudes in response to changing average climatic conditions (Lenoir and Svenning 2015).

However, climate change manifests not only through shifts in mean conditions but also through increased climate variability, leading to changes in the frequency, intensity and duration of extreme climatic events (ECEs) (Seneviratne et al. 2012). ECEs are deviations from typical climate patterns, often characterised by climatic conditions that fall in the tails of historical distributions (van de Pol et al. 2017). They present a unique challenge to ecological systems because species' responses can be shaped by changes in both mean climate and patterns of ECEs, alongside their interactions (Lawson et al. 2015). Indeed, fluctuations in climate variability may have stronger effects on organisms than gradual shifts in average conditions, likely because they suddenly expose individuals to environments that they would have otherwise experienced and adapted to over larger timescales, and thus may exceed their physiological limits (Vasseur et al. 2014).

Global climate predictions indicate a marked increase in the frequency and intensity of ECEs (IPCC 2023), which is already evident worldwide, exemplified by more frequent and prolonged heat waves (Perkins‐Kirkpatrick and Lewis 2020) and intensified extreme precipitation events (Tabari 2020). This escalating trend in ECEs necessitates understanding their ecological ramifications. However, the very nature of ECEs—their rarity and unpredictability—poses significant methodological challenges. Difficulties in eliminating confounding variables and achieving statistically robust assessments have led to a predominant focus on short‐term impacts, leaving the effects of multiple extreme events and long‐term responses understudied (Bailey and van de Pol 2016; Regan and Sheldon 2023).

Despite these challenges, a few studies on animal populations have revealed how ECEs can disrupt life‐histories and drive evolutionary change. Blue tits ( *Cyanistes caeruleus* ) experiencing extremely hot days while rearing nestlings had fewer fledglings and increased selection for earlier breeding (Marrot et al. 2017). Reduced reproductive output was also observed in great tits ( *Parus major* ) that were exposed to multiple ECEs (Regan and Sheldon 2023). Furthermore, survival in red‐winged fairy‐wrens ( *Malurus elegans* ) and white‐browed scrub wrens ( *Sericornis frontalis* ) was more strongly linked to temperature extremes than average conditions, with carry‐over effects of climate in prior seasons mediating size‐dependent mortality (Gardner et al. 2017). In superb fairy‐wrens ( *Malurus cyaneus* ), weather variables exerted counteracting effects on offspring body size across different timescales, illustrating the complexity of disentangling short‐term and cumulative climatic impacts (Kruuk et al. 2015). Yet, most existing research has focussed on the isolated effects of ECEs. There is a pressing need for comprehensive studies that examine how multiple ECEs interact with other ecological factors and long‐term climatic trends to influence wild populations.

Body mass at fledging is frequently used as a fitness proxy in birds. Fledging mass integrates physiological condition, environmental constraints, and parental investment into a single metric. Furthermore, it has been shown to be predictive of survival and reproductive success in many species including great tits (Both et al. 1999; Bouwhuis et al. 2015; Garant et al. 2004; Monrós et al. 2002; Perrins 1965). Temperature fluctuations can significantly influence nestling growth rates, with both positive (Marques‐Santos and Dingemanse 2020; Matthysen et al. 2011; Mccarty and Winkler 1999) and negative (Mainwaring and Hartley 2016) effects observed depending on the context and species. Warmer temperatures may enhance growth by increasing food availability and reducing thermoregulatory costs, but extreme heat can lead to dehydration and reduced parental provisioning. Observational studies of great tits have also shown that increased precipitation during spring leads to lower fledging mass, potentially due to reduced parental foraging effort during rainfall (Keller and Van Noordwijk 1994; Radford et al. 2001). Experimental manipulations of nest microclimates have further demonstrated that higher nest temperatures can both enhance (Dawson et al. 2005) and hinder (Andreasson et al. 2018; Rodríguez and Barba 2016b; Woodruff et al. 2025) growth rates in many cavity‐nesting birds. These seemingly contradictory findings highlight how context‐dependent temperature effects can be. Most of these studies have explored the impacts of average climate, with notable temperature effects during specific developmental windows. Moreover, there is large variation in local climatic conditions among study sites. Temperature rises may exceed species' thermal limits and be more detrimental to growth and survival in warmer regions as compared to cooler habitats.

Given the projected increase in ECEs, understanding their effects on body mass is critical for predicting population‐level responses to climate change. Our study system of great tits in Wytham Woods, Oxfordshire, UK, established in 1947 (Lack 1964), is an appropriate site to examine ecological responses to environmental changes, including climate shifts and rare events, over a timeframe that spans significant variations in ecosystem dynamics. Great tits are passerines that inhabit temperate forests and breed during spring when they rear large broods on an insect diet (Lack 1964). The rearing period of these altricial birds is particularly sensitive to temperature fluctuations, with an ectothermic phase in the initial few days before they eventually attain thermoregulatory mechanisms (Rodríguez and Barba 2016a). They exhibit rapid growth over a well‐defined period, with nestlings growing to 10 times their body mass, from hatching to fledging, in approximately 21 days. This allows for separate analysis of specific developmental phases to understand how variable climates may influence particular stages of nestling growth.

Our long‐term dataset, spanning more than 83,000 individual‐level observations across 60 years analysed here, enabled us to detect rare ECEs, and analyse their impacts on a natural population over ecologically meaningful timescales—an important feature given the rarity and unpredictability of such events. We leveraged this to understand how exposure to ECEs during critical developmental periods may impact both short‐term and long‐term outcomes in this population. To this end, our study had three objectives. First, we assessed the direct effects of ambient weather, and ECEs on fledging mass, at two different developmental stages, hatchling (0–7 days old) and nestling (8–15 days old), which are expected to exhibit differential sensitivities to temperature fluctuations. Second, recognising that climatic factors rarely act in isolation, we explored how ECEs may interact with ambient weather variables and relative laying date, that is how early or late a clutch is laid within each breeding season compared to the seasonal average. These interactions are important for understanding how the impacts of ECEs may be influenced by concurrent variations in temperature, rainfall, or other environmental conditions. Earlier broods in temperate trophic‐matching systems like Wytham generally have access to more abundant resources (e.g., peak caterpillar availability) (Charmantier et al. 2008; Verboven and Visser 1998) and may better withstand environmental stresses, although this relationship is influenced by variation in both food timing and parental capacity. By examining variation in relative lay date and its interaction with exposure to extreme climatic events (ECEs), we assessed how phenological timing among broods within a season may modulate offspring responses to climate variability, which is crucial given that Wytham's great tits have shifted towards earlier laying over the years (Charmantier et al. 2008). Finally, we investigated whether exposure to ECEs during development has an effect on apparent survival to recruit to the breeding population, to explore long‐term consequences of extreme climate variability on population dynamics.

## 2 Methods

### 2.1 Study System

The great tit population in Wytham Woods, Oxfordshire, UK, has been systematically monitored since 1947 (Perrins 1965). In this mixed‐deciduous woodland, over 1000 artificial nestboxes have been provisioned for cavity‐nesting passerines since the 1960s. Wytham uses standardised ‘Schwegler 1B’ woodcrete nestboxes, which have remained the same design since the late 1970s (wooden boxes were used prior to this and were gradually replaced) and exhibit greater thermal instability than natural cavities due to poorer insulation from ambient temperatures (Sudyka et al. 2023), likely exposing nestlings to greater temperature extremes. These nest‐boxes are visited at least once a week during the breeding season (April–June) to collect information on egg‐laying date (date when the first egg is laid), hatching date, clutch size and number of fledglings. As part of our standard protocol, nestlings are fitted with unique metal rings from British Trust for Ornithology and weighed to the nearest 0.1 g at 15 days old, when their mass typically plateaus, providing a reliable measure of fledging mass (Bouwhuis et al. 2015). Parents are captured at the nest when nestlings are between 12 and 14 days, during the provisioning stage, and are individually marked if they have not been previously tagged. For this study, we analysed 60 years of data from 1965 to 2024 for 83,935 individual nestlings (belonging to 11,609 broods), using fledging mass and apparent survival (assessed as recruitment to the breeding population in subsequent years) as variables of interest. Consistent with previous work on this system, we decided to use data from 1965 onwards to account for the population stabilising following the installation of new nestboxes in 1961 (Regan and Sheldon 2023). We only used first clutches for all our analyses, and all our data falls within April–June of each year, consistent with the breeding season of this species.

### 2.2 Characterising ECEs During Developmental Periods

To examine the stage‐specific impacts of climate on nestling development, we focused on two distinct periods prior to the fledging mass measurement at 15 days old—the hatchling stage (0–7 days post‐hatch) and the nestling stage (8–15 days post‐hatch). We chose these periods due to their differential environmental sensitivities and physiological characteristics (Marrot et al. 2017). Newly hatched, altricial birds have not reached homeothermy yet, which begins to develop when they are around 4–6 days old. They are limited in their ability to conserve heat due to their high body surface‐to‐mass ratio and absence of feathers (Rodríguez and Barba 2016b). On the other hand, the later nestling stage is characterised by improved thermoregulation but substantially higher food requirements and rapid growth.

We used daily temperature and precipitation data from the Met Office Hadley Centre datasets for central England (https://www.metoffice.gov.uk/hadobs/) and calculated the average temperature and rainfall during both periods. We also used these data to define ECEs as events falling within the extreme 5% tails of the temperature and rainfall distributions observed over the study period (1965–2024). Specifically, we calculated daily deviations from the monthly means and used the 5th and 95th percentiles to establish cut‐offs (Marrot et al. 2017; Regan and Sheldon 2023). Hot ECEs were defined as days with temperature ≥ +4.52°C above the monthly mean, cold ECEs as ≤ −4.49°C below the monthly mean, and rain ECEs as total rainfall in 24 h ≥ 6.20 mm above the monthly mean. It is important to note that our definition allowed for multiple ECEs to be recorded on consecutive days if the extreme conditions persisted, as each day meeting the criteria was counted as a separate event. Using these thresholds, we quantified the frequency of ECEs during both the hatchling and nestling stages for each individual chick. As a sensitivity analysis, we also recalculated hot and cold ECEs using brood‐specific ±15‐day windows around the developmental period, where deviations were calculated from the long‐term mean temperature of each brood's specific window rather than calendar monthly means; this alternative method yielded highly similar ECE counts and model estimates (Table S15A,B; Figure S2).

In order to explore whether the magnitude of the deviation in climatic conditions from normal is relevant, we also quantified ‘more extreme’ ECEs using the 1% tails of the temperature and rainfall distributions, representing conditions much further from the long‐term mean than the standard 5% definition. Consequently, the much higher thresholds for 1% hot, cold, and rain ECEs were daily temperature ≥ +6.27°C, ≤ −6.27°C, and rainfall ≥ 13.41 mm above the respective monthly means (values calculated for the entire 1965–2024 period).

### 2.3 Statistical Analysis

All analyses were conducted using R version 4.3.3 and linear and generalised linear mixed models were fitted using *lme4* (version 1.1.35.3). We conducted three sets of analyses: (1) Effects of ambient weather and ECEs on fledging mass, (2) interactions of ECEs with ambient weather and birth timing, (3) effects of ECEs on apparent survival. We examined data and model residuals for normality using histograms and *Q*–*Q* plots. Gaussian distribution was assumed for most models unless specified otherwise. We also checked for multicollinearity among model variables, confirming that variance inflation factors remained below 3 (Fox and Weisberg 2018) (using the *performance* package [version 0.12.2]). Plots were constructed with predicted trends based on model estimates using the *ggeffects* package (version 1.5.2). All linear mixed models were fitted using the *lmer* function from the lme4 package, and *p*‐values for fixed effects were obtained from the *lmerTest* package, which computes *t* statistics and approximate denominator degrees of freedom using Satterthwaite's method. For generalized linear mixed models (binomial) fitted with *glmer*, Wald *z*‐statistics were used and associated *p*‐values are based on the standard normal approximation. All model outputs with details of predictors, estimates (*β*), standard errors, test statistics, and confidence intervals, are provided in the Supporting Information.

#### 2.3.1 Effects of Ambient Weather and ECEs on Fledging Mass

Using 60 years of continuous life‐history data, we analysed the fledging mass of 83,935 individual nestlings (median [IQR]; 18.5 g [17.6–19.4 g]). For each of the 11,609 broods, we matched average daily air temperature and rainfall values calculated specifically for the two developmental stages (hatchling and nestling) according to the recorded hatching date, which ensured that the weather predictors accurately reflected the environmental conditions experienced by each nestling during specific developmental windows. We constructed individual‐level linear mixed models for both the developmental periods in which fledging mass was the response variable and the average temperature or rainfall during the relevant period was the main predictor. All models included clutch size and laying date as covariates to account for their well‐established effects on nestling body mass. Non‐independence of offspring raised in the same brood, and of broods raised in the same year and at the same location, was taken into account by fitting random effects of year of birth, brood identity, mother identity and natal nest box.

We expected a nonlinear relationship between temperature and fledging mass, based on exploratory plots and biological reasoning, because many physiological and ecological processes involve thresholds, optima, or plateaus that linear models fail to capture. We used natural cubic splines with 5 degrees of freedom (via the *ns*() function in R's *splines* package [version 4.3.3]) to flexibly model this effect. This approach helps capture curvilinear responses by fitting piecewise cubic polynomials that are joined smoothly at knot points. The choice of 5 degrees of freedom offered a balance between sufficient flexibility and model parsimony, to visualise plausible correlations between average temperature experienced during development and the subsequent body mass at fledging. For a detailed explanation of this methodology, see Harrell (2015). For average rainfall models, a linear model provided a robust fit across the data range. While spline models offered flexibility here, they introduced instability in the fit where data was sparse at higher values.

We fitted separate models for exploring the effects of ECEs during the hatchling or nestling stage, by incorporating the number of ECEs as a fixed effect, along with average temperature, clutch size and laying date as covariates. Each type of ECE was incorporated in separate models due to collinearity between the number of cold and hot ECEs. We addressed potential multicollinearity between lay date and weather variables using path analysis, decomposing total lay date effects into direct and indirect components mediated by temperature and ECEs (Table S10, Figure S1). Lay date, temperature, and ECEs were correlated as expected, but multicollinearity diagnostics (VIFs < 3) and path decomposition confirmed all predictor effects were statistically independent and biologically interpretable. We also constructed a spline model for hot ECEs as it had a better fit than a linear model. Random effects here were the same as before. All fixed effects were scaled to a mean of zero and standard deviation of one to allow for a direct comparison between effect sizes. Furthermore, we conducted two sensitivity analyses to rule out potential carry‐over effects of ECEs across developmental stages. First, we refitted nestling stage ECE models using only individuals that experienced zero ECEs of that type during the hatchling stage. Second, we tested for interactions between the number of ECEs during both the stages. However, nestling stage effects were robust to exclusion of prior‐exposed nestlings, and cross‐stage interactions were small (Table S9).

Additionally, to test for the effects of even more extreme ECEs, we constructed mixed‐effects models within the same framework as described above, using binary indicators for the presence of at least one very extreme event (1% tail) and at least one extreme event (5% tail) during each developmental stage. These stage‐ and event‐type–specific binary variables were derived from the same temperature‐ and rainfall‐based definitions of ECEs, but coded as presence/absence rather than counts. As before, models were fitted separately for hot, cold, and rain ECEs at each stage using the same fixed covariates (average temperature for the relevant stage, laying date, clutch size) and random intercepts (birth year, brood identity, mother identity, natal nest box).

#### 2.3.2 Interactions of ECEs With Ambient Weather and Birth Timing

To examine how ambient climatic conditions influence nestling mass within the context of extreme climatic events (ECEs), we expanded our modelling framework by introducing interaction terms between weather variables and ECE metrics. Specifically, we tested whether the effects of average temperature vary with the presence and severity of rain ECEs, and whether the influence of average rainfall changes in the presence of hot or cold extremes. This was designed to reflect the ecological reality that climate variables seldom operate independently, and that the biological impacts of extreme climate can be shaped by prevailing temperature and precipitation patterns.

Furthermore, to assess the interplay between ECEs and resource availability, we incorporated relative lay date, calculated as the difference between individual lay date and the population average in the same year, as an interaction term with the number of ECEs. We acknowledge that resource availability is a complex process influenced not only by laying date, but also factors like prey phenology, weather, habitat quality, and parental capacity, which are challenging to quantify directly at this scale. Nevertheless, we used relative lay date as a proxy for seasonal shifts in resource dynamics, because earlier laying generally aligns with peak food abundance and can potentially afford parents greater flexibility in matching offspring development to resource peaks (Simmonds et al. 2017), particularly in systems like Wytham that rely on trophic matching (Charmantier et al. 2008; Verboven and Visser 1998). This approach allowed us to investigate whether the timing of breeding within the season changes the degree to which nestlings are affected by extreme climatic variability. All interaction models used ECE variables with the 5% threshold. We also quantified the absolute temperature and rainfall associated with ECE exposure for early (≤ 15th percentile relative lay date within each cohort year) and late (≥ 85th percentile) broods across both developmental stages (Tables S11–S13) to document the actual environmental conditions experienced by nestlings across the breeding season.

#### 2.3.3 Effects of ECEs on Apparent Survival

Apparent survival for each individual was determined by assessing the local recruitment of the fledgling to the breeding population in subsequent years. Local recruitment was thus a binary variable with 1 for recruited and 0 if not known. This measure underestimates actual survival because birds do emigrate from Wytham, or may breed locally without being captured, but emigration has been shown to be independent of fledging mass and unlikely to bias results (Bouwhuis et al. 2015). We analysed 83,935 individuals hatched between 1965 and 2024, with the latest recruitment to the 2025 breeding season.

To investigate the long‐term effects of ECEs during the hatchling and nestling stages, we constructed generalised linear mixed models (GLMM) with local recruitment as the response and the number of ECEs (5% threshold) as the fixed effect. As before, average temperature and clutch size were incorporated as additional fixed effects, with birth year, brood identity, mother identity, and nest box as random effects. The GLMMs assumed a binomial distribution. We also constructed additional models where the laying date was accounted for as a fixed effect.

For these models, we enhanced the predictive power to examine ECE effects by combining higher ECE frequencies into a single category, treating the number of ECEs as a categorical variable rather than a continuous one (Table S7). This accounted for the rarity of individuals experiencing very high ECE frequencies and allowed us to detect significant effects of ECEs beyond a certain threshold (Regan and Sheldon 2023).

## 3 Results

### 3.1 Effects of Ambient Weather and ECEs on Fledging Mass

**FIGURE 1:**
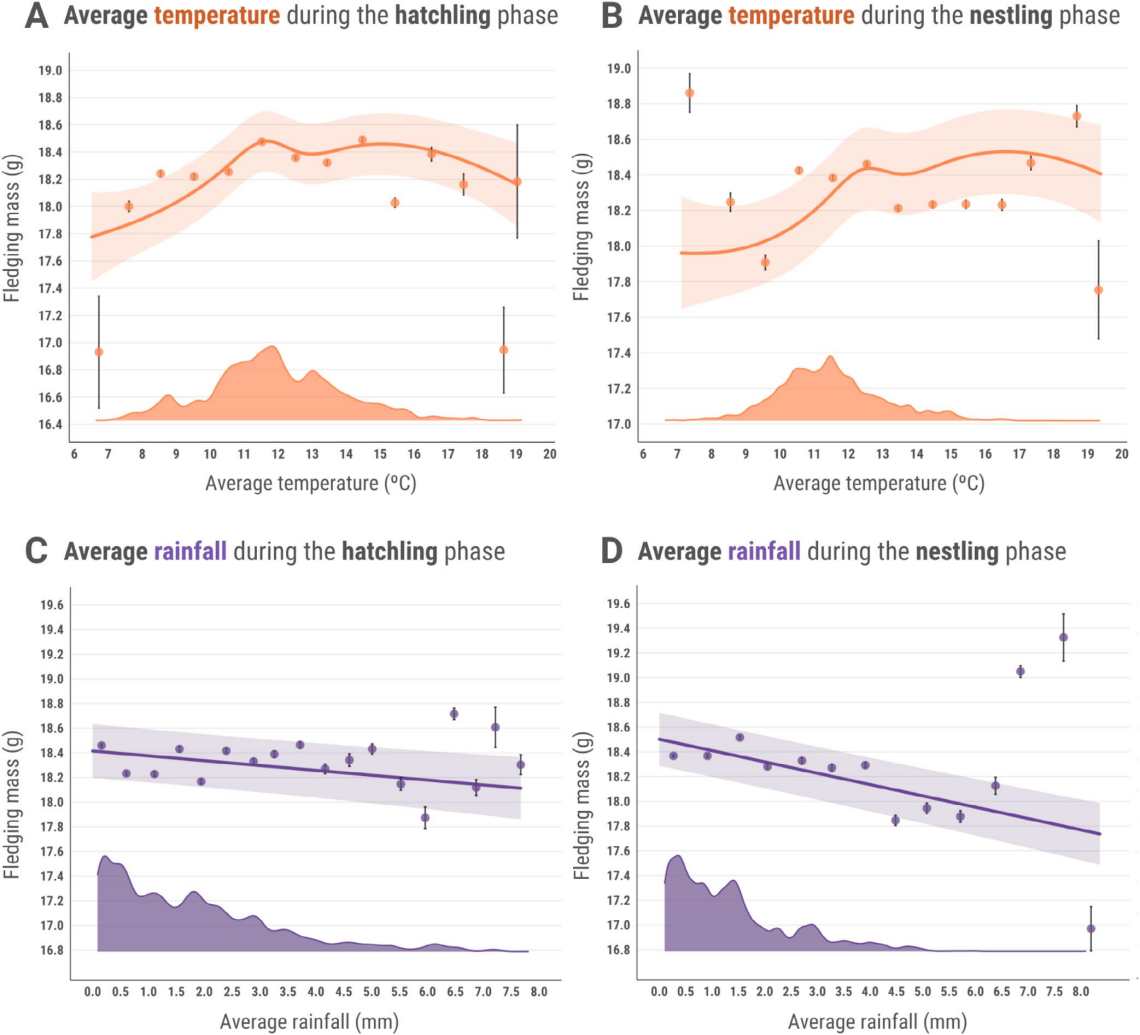
Associations between average temperature (°C) and fledging mass (g) in the hatchling (A) and nestling (B) stages, and between average rainfall (mm) and fledging mass (g) in the hatchling (C) and nestling (D) stages, for great tits (*Parus major*) in Wytham Woods, UK, from 1965 to 2024. Solid lines and associated ribbons indicate predicted trends and 95% confidence intervals from the models. The coloured dots and lines represent the average fledging mass ± standard error from observed data grouped in 1°C (temperature in orange) and 0.5 mm (rainfall in purple) bins respectively. The density plots above the *x* axes show the distribution of data points from observed data (*N* = 83,936 individuals).

Linear mixed models revealed significant non‐linear relationships between average ambient temperature and fledging mass, best described by natural cubic splines (Table S1.1). In the hatchling phase, three of five spline basis functions were highly significant (*β*_1_ = 0.82 ± 0.15, *t*_(9863)_ = 5.33, *p* < 0.001, *β*_2_ = 0.60 ± 0.18, *t*_(9878)_ = 3.35, *p* < 0.001, *β*_3_ = 0.79 ± 0.13, *t*_(10,060)_ = 6.26, *p* < 0.001; Figure 1A, Table S1), flexibly capturing inflections beyond simple quadratic patterns. Similarly, three spline terms significantly predicted fledging mass in the nestling stage, although not as strongly as in the hatchling stage (*β*_1_ = 0.50 ± 0.18, *t*_(9981)_ = 2.72, *p* = 0.006, *β*_2_ = 0.37 ± 0.2, *t*_(9983)_ = 1.82, *p* = 0.06, *β*_3_ = 0.84 ± 0.15, *t*_(10,220)_ = 5.42, *p* < 0.001; Figure 1B, Table S1). High average rainfall during both phases was associated with reduced fledging mass, with the effect being more pronounced in the nestling phase (hatchling: *β* = −0.063 ± 0.018, *t*_(10,260)_ = −3.467, *p* < 0.001; nestling: *β* = −0.143 ± 0.015, *t*_(10,250)_ = −9.109, *p* < 0.001; Figure 1C,D).

**FIGURE 2:**
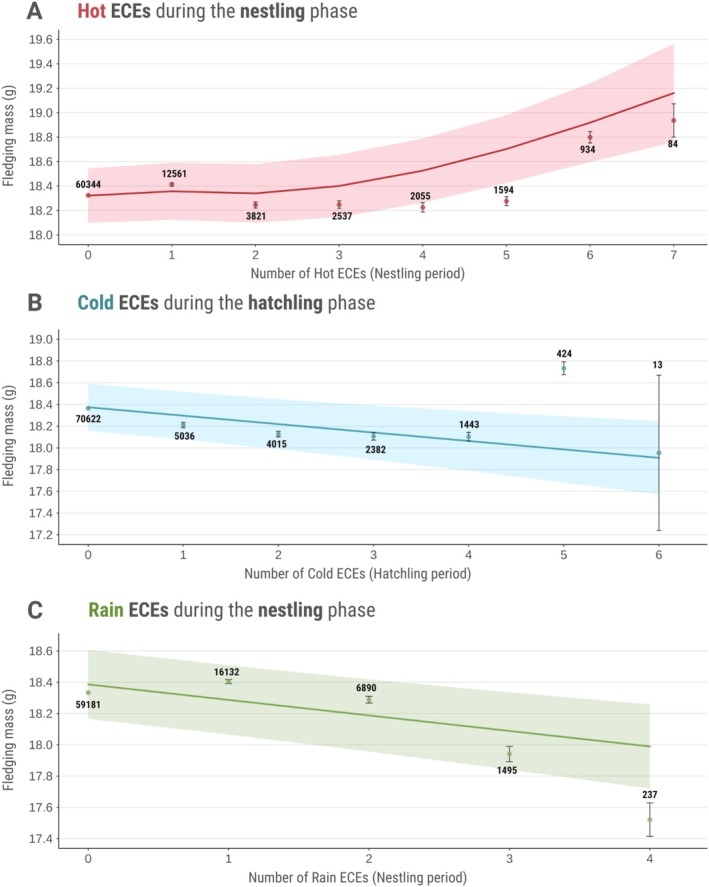
Predicted effects of (A) hot extreme climatic events (ECEs) in the nestling phase, (B) cold ECEs in the hatchling phase, and (C) rain ECEs in the nestling phase on fledging mass (g), for great tits ( *Parus major* ) in Wytham Woods, UK, from 1965 to 2024. Solid lines and associated ribbons indicate predicted trends and 95% confidence intervals from the models. The coloured dots and lines represent the average fledging mass ± standard error for each frequency of ECE from observed data (*N* = 83,936 individuals). Predicted trends account for the model structure, incorporating fixed and random effects, which may cause predicted values to differ from raw summary statistics. Sample sizes for each frequency of ECE are indicated above the corresponding data points.

Hot ECEs during the hatchling stage had no significant effect on fledging mass (*β* = −0.022 ± 0.023, *t*_(10,170)_ = −0.99, *p* = 0.32). In contrast, fledging mass increased nonlinearly with increased frequency of hot extreme events during the nestling stage, as indicated by a natural spline model (Figure 2A). Predicted fledging mass remained relatively stable up to three hot days before increasing more sharply with further exposure. Nestlings that had experienced seven hot ECEs were predicted to weigh approximately 0.5 standard deviations (4.5%) more than those with no exposure. These hot ECE effects remained significant alongside strong covariates including relative lay date (*β* = −0.223, *t* = −15.4, *p* < 0.001), which measures how early/late clutches are laid relative to the seasonal average. Early‐laid nestlings naturally encounter cooler temperatures and fewer hot days. However, path analysis showed that weather mediates only a third of lay date's total effect on fledging mass (Figure S1). Hot ECEs, thus, operate largely independently of these seasonal correlations and a substantial portion of lay date effects on fledging mass likely stem from non‐weather‐related factors.

In contrast to the effects of hot ECEs, frequent cold ECEs during the hatchling period were associated with a significant reduction in fledging mass (Figure 2B). The linear model predicted that individuals experiencing cold ECEs throughout the entire hatchling period (i.e., 6 consecutive days) would fledge at weights that were on average 0.28 standard deviations (SD) lower than those of hatchlings without cold ECE exposure. No significant effect of rain ECEs was observed during the hatchling stage (*p* = 0.14), but the nestling stage showed vulnerability to extreme precipitation (*β* = −0.073 ± 0.016, *t*_(10,270)_ = −4.536, *p* < 0.001), with a predicted average reduction of 0.4 g (0.24 SD) in fledging mass of nestlings that experienced 4 days of extreme rainfall compared to those with no rain ECEs (Figure 2C).

Additional models using binary indicators for the presence of at least one very extreme event (1% threshold) yielded patterns broadly consistent with the frequency‐based models above, albeit with mostly non‐significant and weaker effect sizes across developmental stages, especially for hot and cold ECEs. Very extreme rainfall across both hatchling (*β* = −0.041 ± 0.016, *t*_(10,160)_ = −2.578, *p* = 0.009) and nestling (*β* = −0.042 ± 0.01, *t*_(10,200)_ = −2.488, *p* = 0.013) stages was associated with reduced fledging masses, although these effects were somewhat weaker than those observed for the 5% rain ECEs. Full details of all count‐based 5% ECE and binary 1% ECE model results are provided in Tables S2 and S3.

### 3.2 Interactions of ECEs With Ambient Weather and Birth Timing

**FIGURE 3:**
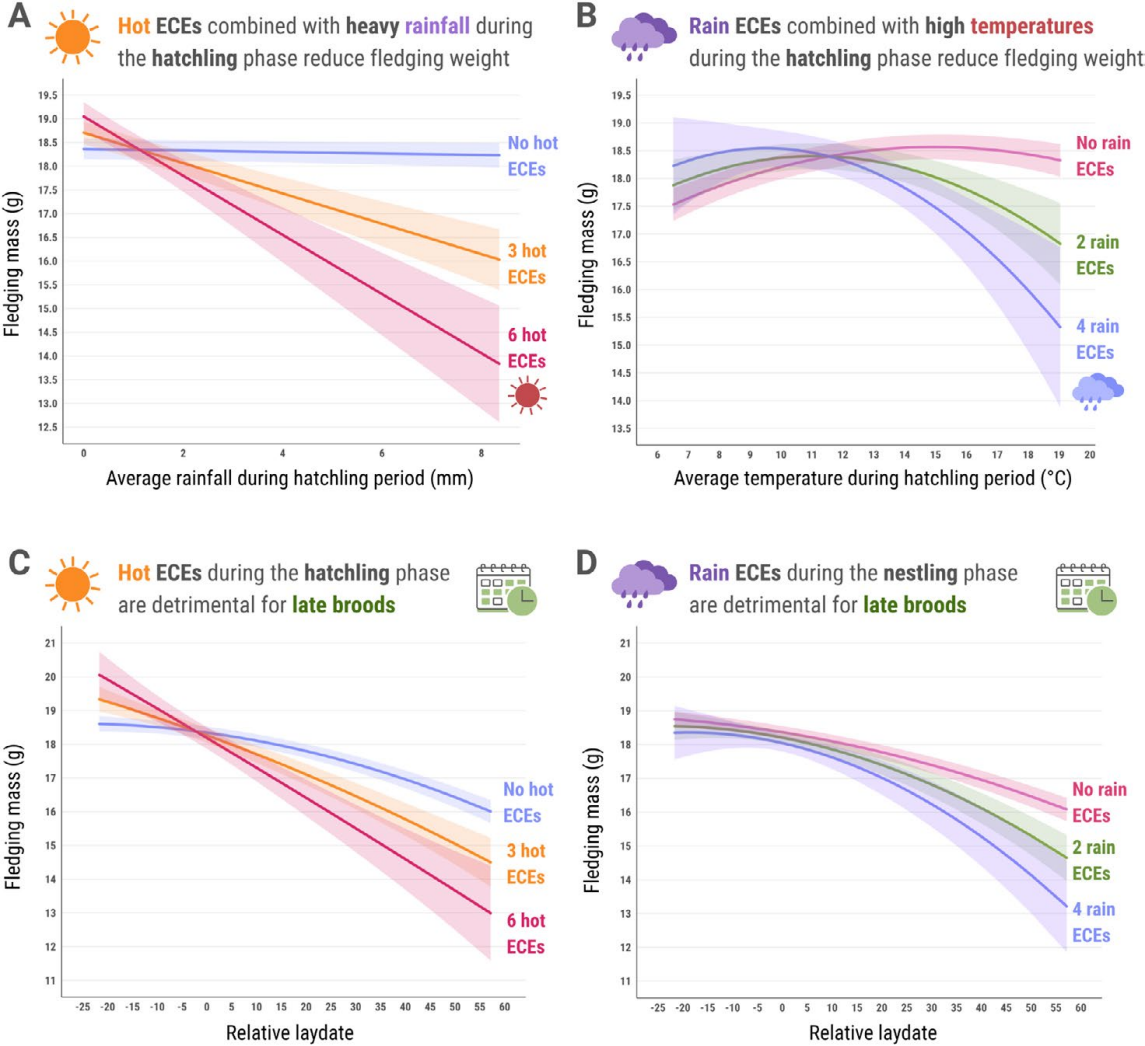
Predicted trends from interactions between (A) number of hot extreme climatic events (ECEs) × average rainfall (mm) during the hatchling stage, (B) number of rain ECEs × average temperature (°C) during the hatchling stage, (C) number of hot ECEs during the hatchling stage and relative lay date, (D) number of rain ECEs during the nestling stage and relative lay date, for great tits ( *Parus major* ) in Wytham Woods, UK, from 1965 to 2024. Relative lay date is the difference between individual lay date and average lay date of the population in that year; negative values indicate relatively earlier clutches. Solid lines and associated ribbons indicate predicted trends and 95% confidence intervals from the models.

Our models revealed significant interactive effects between the frequency of ECEs and ambient environmental conditions on nestling growth outcomes, but only during the hatchling stage. While experiencing rain and hot ECEs during this early phase of development did not lead to any significant direct effects on fledging mass, their combined effects with ambient weather variables were pronounced. In particular, the detrimental impact of increased precipitation during this stage was amplified with increasingly frequent hot ECEs (hot ECEs × mean rainfall: *β* = −0.162 ± 0.022, t_(10,230)_ = −7.367, *p* < 0.001; Figure 3A). For example, at the highest frequency of hot ECEs (6 events), predicted fledging mass decreased by approximately 27%, corresponding to a 3.15 SD reduction as average rainfall increased from 0 to 8.36 mm. In contrast, nestlings with no exposure to hot ECEs showed a decrease of only 0.08 standard deviations (0.7%) (Figure 3A).

Furthermore, extreme precipitation during this stage narrowed the optimal temperature range for early development, exacerbating the negative effects of high temperatures when co‐occurring (rain ECEs × mean temperature: *β* = −0.085 ± 0.017, *t*_(10,580)_ = −4.973, *p* < 0.001; Figure 3B). Increasing rain ECEs from 0 to 4 at high average temperatures (~19°C) led to about a 16% reduction in predicted fledging mass (−1.817 SD). These results highlight how combined stressors can synergistically influence thermoregulatory mechanisms in hatchlings.

Stage‐specific interactions between extreme climatic events (ECEs) and environmental conditions were further modulated by hatch timing relative to the population's seasonal average. Predicted fledging masses from our models revealed that extreme heat exposure during the hatchling stage may disproportionately impact relatively later broods, despite similar absolute temperatures during frequent hot ECEs (Figure 3C, Table S11). Estimates for the earliest broods (~22 days earlier than the seasonal average) predicted a moderate increase in fledging mass of about 7.8% (+0.88 SD) for individuals that have not experienced any hot ECEs, compared to those that have experienced them throughout the hatchling stage. In stark contrast, nestlings from later broods (~57 days after the seasonal mean) showed a predicted decline of nearly 1.83 standard deviations (18.9%) under equivalent thermal extremes. Very early broods experienced average baseline temperatures of 10.8°C rising to 16.5°C during maximum exposure (6+ hot ECEs), while later broods went from 12.5°C to 16.8°C (Table S11). Despite these similar thermal conditions, a predicted difference of approximately 4.27 SD (> 35%) in fledging mass was observed between early and late broods exposed to six consecutive days of extreme heat (hot ECEs × relative lay date: *β* = −0.067 ± 0.012, *t*_(10,070)_ = −5.181, *p* < 0.001; Figure 3C).

Similarly, rain ECEs during the nestling phase had a greater negative impact on broods born later in the breeding season. This interaction widened the fledging mass gap between early and late clutches. In the absence of rain ECEs, the predicted fledging mass difference between earliest and latest clutches was 1.61 SD (14.2%), but this disparity nearly doubled to 3.1 SD (27.9%) if nestlings experienced 4 rain ECEs (Figure 3D). The accelerating decline in late broods with increasing rain ECEs is reflected in a significant quadratic interaction term (*β* = −0.014 ± 0.006, *t*_(10,550)_ = −2.334, *p* = 0.01).

### 3.3 Effects of ECEs on Apparent Survival

**FIGURE 4:**
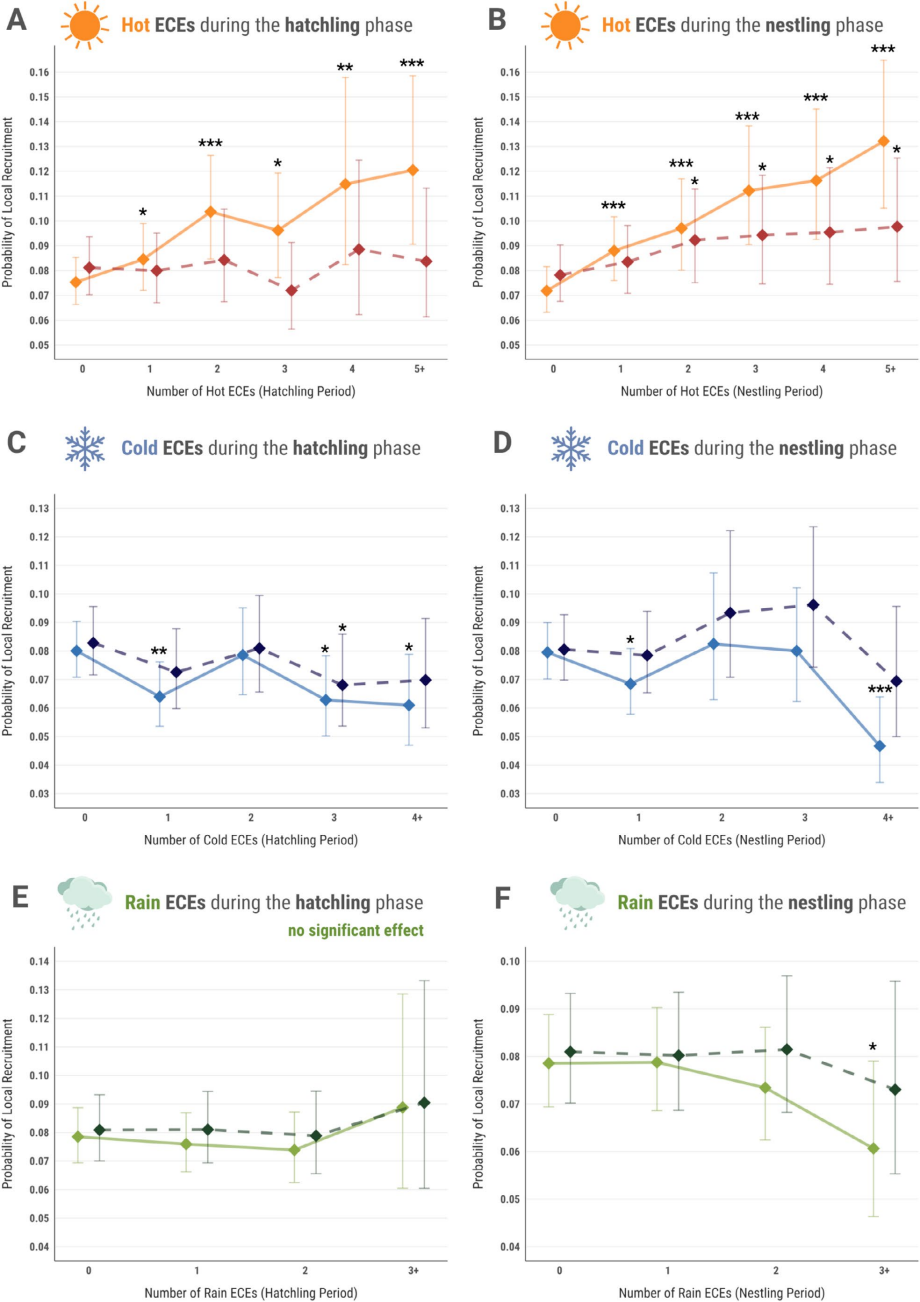
Associations between apparent survival probability (0 to 1) and (A) number of hot extreme climatic events (ECEs) during the hatchling stage; (B) number of hot ECEs during the nestling stage; (C) number of cold ECEs during the hatchling stage; (D) number of cold ECEs during the nestling stage; (E) number of rain ECEs during the hatchling stage; (F) number of rain ECEs during the nestling stage, for great tits ( *Parus major* ) in Wytham Woods, UK, from 1965 to 2024. Shown are model predictions and associated credible intervals; *N* = 82,229 individuals. Solid lines represent predicted trends before laying date was accounted for in the model, whilst dashed lines correspond to model predictions when individual laying dates were included as fixed effect. Significant effects are marked with an asterisk, and indicate the comparison of each level with 0 ECEs (***p < 0.001, **p < 0.01, *p < 0.05).

We found evidence that recruitment probability (a proxy for survival) declined with increased exposure to high frequencies of extreme cold and rain during hatchling and nestling phases. Data from 83,935 individuals (1965–2024) revealed an average recruitment probability of 9.1% over the study period. Exposure to cold ECEs during the hatchling stage reduced survival probability, with a significant negative effect observed for individuals experiencing a single extremely cold day (*β* = −0.241 ± 0.07, *z* = −3.273, *p* = 0.001). Survival probability was predicted to decrease by over 25%, from 8.0% for individuals with no cold ECE exposure to 5.8% for those exposed to four or more cold ECEs (*β* = −0.30 ± 0.128, *z* = −2.368, *p* < 0.05, Figure 4C). The impact of 4+ days of extreme cold on survival probability was significantly stronger during the nestling stage (*β* = −0.56 ± 0.157, *z* = −3.597, *p* < 0.001). Survival probability was predicted to decrease by > 40%, from 7.7% for individuals that did not experience any cold ECEs to 4.5% for those exposed to four or more cold ECEs (Figure 4D). Rain ECEs during both the stages, especially during early development, had negligible effects on apparent survival (*p* > 0.1, Figure 4E,F). However, a marginally significant negative effect was observed for individuals that experienced three or more days of extreme rain (*β* = −0.275 ± 0.128, *z* = −2.145, *p* < 0.05, Figure 4F).

Experiencing hot ECEs during both the hatchling and nestling stages, however, appeared to confer a survival advantage, with models predicting significant positive associations between exposure to hot days during development and recruitment probability. For hatchlings, survival probability increased significantly with exposure to hot ECEs, with a 25% increase for individuals experiencing two or more extreme heat events (*β* = 0.345 ± 0.094, *z* = 3.649, *p* < 0.001). The effect was strongest for those exposed to five or more hot ECEs, where survival probability showed a notable 50% increase (*β* = 0.522 ± 0.15, *z* = 3.474, *p* < 0.001, Figure 4A). Similarly, for nestlings, survival probability increased progressively with higher frequencies of hot ECEs. Individuals exposed to five or more extreme heat events during this stage exhibited the strongest positive association, with recruitment likelihood increasing by 85.7% (*β* = 0.712 ± 0.121, *z* = 5.883, *p* < 0.001, Figure 4B).

However, when lay date was included in the models, the effects of all ECEs (hot, cold, and rain) on recruitment probability were attenuated, with most estimates decreasing in magnitude and several effects losing statistical significance (Table S6, Figure 4), indicating that lay date is the dominant seasonal driver of survival outcomes.

## 4 Discussion

With observed climate patterns increasingly aligning with long‐term projections, and with extreme climatic events (ECEs) becoming more frequent and intense (IPCC 2023), understanding their ecological impacts has become a growing priority. Our study provides a comprehensive analysis of the impacts of ECEs during sensitive developmental phases on the growth of great tit nestlings and their subsequent survival to adulthood, offering critical insights into how climate variability can influence natural populations.

We found that the hatchling stage (0–7 days post hatching) exhibited heightened sensitivity to temperature changes compared to the nestling stage (8–15 days post hatching), as revealed by stronger non‐linear responses to average temperature during this period (multiple significant spline terms, Table S1), likely due to limited thermoregulatory capability. During early development, hatchlings lack well‐developed feathers and have not developed homeothermy, leaving them poorly equipped to maintain thermal stability. Exposure to extreme cold during this period was also particularly detrimental to growth. During prolonged extreme cold conditions, hatchlings are likely suffering hypothermia‐associated declines, such as slowed metabolism, in addition to diverting energy toward thermoregulation at the expense of growth and development (Dawson et al. 2005)—a capacity they possess only to a very limited extent to begin with. Unlike winter‐acclimatised great tit adults, which elevate basal metabolic rates and adjust respiration to enhance cold tolerance (Bech and Mariussen 2022), hatchlings lack such physiological adjustments, amplifying vulnerability. Furthermore, increased parental brooding effort, albeit crucial for hatchling survival in such situations, may additionally limit food availability due to reduced foraging activity (Rodríguez and Barba 2016a).

The nestling stage, on the other hand, marked with increased energetic demands of the growing chicks, was more strongly influenced by precipitation patterns (both average and extreme) and extreme heat events. While rain ECEs and elevated average rainfall reduced fledging mass, hot ECEs enhanced growth—a contrast likely mediated by their opposing indirect effects on food availability. Heavy rainfall can deter birds from foraging and can also dislodge caterpillars and other insect prey from vegetation, making them harder to spot by parents (Radford et al. 2001). Conversely, heat can increase insect activity and visibility, potentially boosting prey availability (Mccarty and Winkler 1999; Schöll et al. 2016). Furthermore, if nestlings are sufficiently warm, parents can dedicate more time to foraging and provisioning, rather than brooding (Dawson et al. 2005; Rodríguez and Barba 2016b). In our temperate study area, average temperatures experienced by broods exposed to 6–7 consecutive hot ECEs are 16.5°C–17°C (Table S14A). While warmer than baseline ambient conditions (0 hot ECEs), these temperatures represent moderate rather than extreme heat, and are well within great tit thermal tolerance. Nestlings can therefore benefit from reduced thermoregulatory costs (Andreasson et al. 2018), and capitalise on increased prey abundance associated with these warmer conditions, promoting their rapid growth (Dawson et al. 2005). Additionally, great tit nestlings primarily consume caterpillars, which have high water content, further reducing their risks of dehydration (Andreasson et al. 2018). These habitat‐specific dynamics illustrate how beneficial hot ECEs in temperate climates may contrast sharply with their detrimental impacts in hotter regions (Rodríguez and Barba 2016b).

Possibly, this temperate climate context may also help explain why our analyses of the rarest and most extreme events, defined by the 1% tails of temperature and rainfall distributions, showed generally weaker and mostly non‐significant effects for temperature extremes. Despite such high thresholds, the maximum daily temperature for a 1% hot ECE was around 28°C. While this would qualify as a heatwave in central England, its ecological impact may be modest compared to tropical or arid systems, or even Mediterranean habitats where this species experiences more severe heatwaves exceeding 35°C (Rodríguez and Barba 2016b). In contrast, very extreme rainfall events, though also rare, represent more severe deviations from typical conditions in our study area (with peak rainfall reaching up to 40 mm), and were linked to reductions in fledging mass across developmental stages, albeit with smaller effect sizes than the more frequent 5% rain ECEs. However, the rarity of these 1% events inherently limits statistical power, making it difficult to reliably detect their impacts even with a robust dataset.

Apart from isolated effects we found that ECEs can interact with ambient environmental conditions to collectively influence nestling growth outcomes. During the hatchling stage, while rain and hot ECEs did not directly affect growth, their interactions with mean climatic variables proved significant. The combination of extreme rainfall and higher average temperatures, or the converse of extreme heat combined with consistently higher precipitation, predicted a particularly challenging scenario for early development. These synergistic effects likely stem from the limited thermoregulatory capabilities of young hatchlings, exacerbated by the resource limitations imposed by prey unavailability and altered parental foraging behaviour during extreme rainfall. Increased temperatures combined with high humidity could also favour greater abundances of nest‐dwelling ectoparasites, which have well‐documented negative effects on nestling condition and growth (Dube et al. 2018; Dudek et al. 2021; Pryor and Casto 2015). However, several nest‐temperature manipulation studies report that warmer nest microclimates can actually reduce ectoparasite loads, likely via desiccation of parasites and their larvae, suggesting that ectoparasitism is unlikely to be a primary mechanism underlying reduced hatchling growth under hot ECEs in our system (Castaño‐Vázquez et al. 2021, 2022, 2025; García‐del Río et al. 2025). Interestingly, nestlings showed a contrasting pattern, where the negative effect of higher rainfall was reversed in the presence of extreme heat, although this interaction was marginally significant (Table S4). This unexpected result suggests that older nestlings might be better equipped to handle, and even benefit from, the combination of warmth and moisture, unlike hatchlings, which were predicted to be especially vulnerable to compounded climatic stressors.

Perhaps most importantly, our findings further demonstrate that the consequences of ECEs on nestling growth outcomes are highly contingent upon seasonal breeding phenology within the Wytham great tit population. Being part of early clutches confers significant advantages, as abundant food resources earlier in the season may support better growth outcomes. Great tits in the Wytham population have been shown to use spring temperature cues to adaptively adjust their incubation onset after laying has already begun, to synchronise their offspring's peak energetic demands with peak food availability (Simmonds et al. 2017). Earlier laying thus allows parents greater flexibility over incubation timing and consequently optimises provisioning opportunities for their nestlings, as compared to late breeders who have missed this optimal timing window. Consequently, our models indicated that broods laid earlier in the season demonstrate a degree of resilience or even benefit from hot ECEs during the hatchling stage, whereas late broods face significantly greater challenges. This pattern persists despite hot ECEs producing similar absolute temperatures across seasonal timings (Table S11). Early broods likely benefit from hot ECEs because they provide warmer conditions during cold springs, which are more favorable for development, especially when food resources are abundant early in the season. In contrast, the same temperatures exacerbate the disadvantages of being part of a late brood, where reduced food availability combined with high temperatures may be particularly detrimental to growth. Similarly, rain ECEs during the nestling stage possibly intensify resource limitations for late‐clutch offspring, by further reducing foraging opportunities for already scarce prey late in the season.

While ECEs can exacerbate poor resource availability and challenging climatic conditions, their impacts in isolation are not extremely strong in this population. As our results show, nestlings from early broods may even benefit from higher temperatures, experiencing them as moderate warmth rather than extreme heat. However, while breeding earlier within a season may help offset the negative impacts of ECEs, longer‐term shifts in breeding phenology add further complexity to population responses. Over the past few decades, our study population has shifted to earlier breeding, likely as an adaptive response to warming temperatures (Charmantier et al. 2008). This has inadvertently increased exposure to extreme cold events early in the breeding season (Table S12; Regan and Sheldon 2023), which our findings show to be damaging to nestling growth. Consequently, although earlier breeding within a season may mitigate some challenges, the overarching trend toward earlier laying could paradoxically increase the population's susceptibility to detrimental cold extremes. This suggests that adaptive shifts in phenology may not entirely eliminate the risks posed by evolving extreme climatic conditions, emphasising the need for continuous monitoring to assess how future climate scenarios affect population resilience. Fledging mass in the Wytham great tit population has also gradually declined over the past several decades, driven primarily by increased intra‐ and inter‐specific competition due to increased density rather than temperature changes (López‐Idiáquez et al. 2026). More importantly, selection against smaller nestlings has strengthened over time, suggesting that even small growth deficits from ECEs like extreme cold or rain now have larger and more detrimental consequences for post‐fledging survival than they did in the past.

Our analysis also revealed weak long‐term effects of ECEs, with extreme cold and rain during the nestling period reducing the likelihood of recruiting to the breeding population, likely due to thermal stress and resource depletion. Previous studies have also documented the negative impacts of increased rainfall during critical developmental periods on offspring survival (Arct et al. 2025; Pipoly et al. 2013; Schöll and Hille 2020). Conversely, survival to adulthood was predicted to be more likely if nestlings experienced hot ECEs throughout their development. These findings align with an experimental study on blue tits with artificially elevated nest temperatures (Andreasson et al. 2018). Higher ambient temperatures during the nestling stage were shown to be associated with a higher number of recruits in a wild population of collared flycatchers ( *Ficedula albicollis* ) in a recent study (Arct et al. 2025). These long‐term outcomes may reflect developmental carryover effects, where early‐life thermal environments prime or constrain adult thermoregulatory capacity via environmental matching or growth limitations (Nord and Giroud 2020; Persson et al. 2024). However, no effects of heat treatments on post‐fledging survival were observed in another experiment with great tits (Rodríguez and Barba 2016b). Accounting for the relative egg‐laying timing in our models diminished the effects of ECEs on apparent survival, suggesting that selection pressures favouring earlier clutches may have enhanced resilience to long‐term ECE impacts. However, while earlier breeding may have buffered this population against some climatic challenges, it may not always be protective as global temperatures continue to rise. For example, while hot ECEs currently have limited negative impacts in this temperate population—likely because they do not exceed thermal thresholds (Rodríguez and Barba 2016b)—future increases in heat intensity could impose significant stress on nestlings.

Despite these insights, our study has limitations that warrant consideration. First, our definition of ECEs assumes that their effects have remained constant over a 60‐year period. Shifting climate norms and increased variability mean that past ‘extreme’ events may now be more common, potentially altering their ecological significance over time. Second, we focused on fledging mass and recruitment probability as key metrics but lacked data on intermediate developmental stages where compensatory mechanisms might occur (Sauve et al. 2021). Furthermore, our study used broad‐scale weather data, assuming uniform ambient climatic conditions across all nests, which overlooks the possible effect of fine‐scale habitat heterogeneity. Fine‐scale microenvironmental variation could lead to different ECE exposures and impacts, even for broods in relatively close proximity. For instance, urban great tit nestlings appear less vulnerable to heat than forest ones (Pipoly et al. 2022), suggesting habitat context modulates thermal tolerance. On the same note, while our study identified significant impacts of precipitation on nestling growth, we relied on daily rainfall data that may not capture fine‐scale temporal dynamics. Consecutive days of extreme rain likely have different ecological consequences than intermittent heavy rainfall events, as do associated wind patterns, which can affect aerial insect availability. Future studies should incorporate high‐resolution rainfall and wind data to better understand how temporal weather patterns influence food availability and parental behaviour.

Finally, it is important to consider the broader implications of our findings for other populations. Are the patterns we observed in Wytham Woods generalisable, or are they specific to this population due to its unique environmental context and evolutionary history? Our temperate climate context, where hot ECEs enhance growth unlike in hotter habitats (Rodríguez and Barba 2016b; Pipoly et al. 2022), likely contributes to these dynamics, alongside evolved thermal tolerances shaped by developmental plasticity. This aligns with a growing body of work showing how early‐life thermal conditions can prime adult thermoregulation when matched to later environments or constrain it through growth costs (‘environmental matching’ or ‘silver spoon’ hypotheses; Broggi et al. 2022; Nord and Giroud 2020; Persson et al. 2024). Our findings contribute to this understanding by emphasising the complexity of ecological responses, the importance of developmental stage‐specific vulnerabilities, and the role of phenological shifts in mitigating climate impacts. Understanding the consistency of these stage‐specific responses, microclimate effects, and physiological carryover across different populations and species is crucial for predicting climate change impacts and adaptation potential (Swanson et al. 2022). Future research should therefore focus on incorporating finer‐scale environmental data, investigating the physiological mechanisms underlying the observed responses, and exploring how microclimates and individual variation buffer against extreme events. Ultimately, as ECEs become more frequent and severe, understanding these dynamics will be essential for informing conservation strategies and predicting population‐level responses in a rapidly changing world.

## Author Contributions

All authors contributed to the conceptual development of this study. Devi Satarkar conducted the statistical analyses with substantial inputs on methodology from David López‐Idiáquez and Ben C. Sheldon. Devi Satarkar drafted the manuscript with David López‐Idiáquez, Irem Sepil and Ben C. Sheldon providing critical feedback. All authors approved the final manuscript.

## Conflicts of Interest

The authors declare no conflicts of interest.

## Supporting information


**Table S1.** Ambient climate models: outputs of linear mixed models for fledging mass with ambient climate measures (mean temperature/mean rainfall) during specific developmental stages (hatchling/nestling) as explanatory variables along with lay date, and clutch size as fixed effects. Year of birth, brood identity, mother identity and natal nest box are included as random effects. All fixed effects are scaled to a mean of zero and standard deviation of one. Significant terms (*p* < 0.05) are in bold.
**Table S1.1.** Model selection for ambient temperature effects on fledging mass (based on AICc).
**Table S2.** Extreme climate events (frequency) models: outputs of linear mixed models for fledging mass with number of ECEs during specific developmental stages (hatchling/nestling) as explanatory variables along with mean temperature during the relevant stage, lay date, and clutch size as fixed effects. Year of birth, brood identity, mother identity and natal nest box are included as random effects. All fixed effects are scaled to a mean of zero and standard deviation of one. Significant terms (*p* < 0.05) are in bold. Here, ECEs are calculated with a 5% threshold.
**Table S3.** Extreme climate events (binary) models: outputs of linear mixed models for fledging mass with presence of at least 1 ECE (1% or 5%) during specific developmental stages (hatchling/nestling) as explanatory variables along with mean temperature during the relevant stage, lay date, and clutch size as fixed effects. Year of birth, brood identity, mother identity and natal nest box are included as random effects. All fixed effects are scaled to a mean of zero and standard deviation of one. Significant terms (p < 0.05) are in bold.
**Table S4.** Interaction models (ambient climate × ECE frequency): outputs of linear mixed models for fledging mass with number of ECEs during specific developmental stages (hatchling/nestling) interacting with relevant ambient climate measures as predictors, along with lay date and clutch size as fixed effects. Year of birth, brood identity, mother identity and natal nest box are included as random effects. All fixed effects are scaled to a mean of zero and standard deviation of one. Significant terms (*p* < 0.05) are in bold. Here, ECEs are calculated with a 5% threshold.
**Table S5.** Interaction models (relative lay date × ECE frequency): outputs of linear mixed models for fledging mass with number of ECEs during specific developmental stages (hatchling/nestling) interacting with relative lay date as predictors, along with mean temperature and clutch size as fixed effects. Year of birth, brood identity, mother identity and natal nest box are included as random effects. All fixed effects are scaled to a mean of zero and standard deviation of one. Significant terms (*p* < 0.05) are in bold. Here, ECEs are calculated with a 5% threshold.
**Table S6.** Local recruitment models: outputs of generalised linear mixed models for recruitment probability with number of ECEs during specific developmental stages (hatchling/nestling) as predictors, along with clutch size as fixed effect. Outputs of additional models with lay date as fixed effect have also been detailed below. ECEs are treated here as categorical variables. Year of birth, brood identity, mother identity and natal nest box are included as random effects. All fixed effects are scaled to a mean of zero and standard deviation of one. Significant terms (*p* < 0.05) are in bold. Here, ECEs are calculated with a 5% threshold.
**Table S7.** Higher frequencies of ECEs were grouped into a single categorical level, due to the reduced number of individuals experiencing high frequencies of ECEs. Local recruitment models from Table S6 used categorical ECE variables for analysis. Blue shaded areas indicate the values that were combined for each type of ECE in each developmental period.
**Table S8.** Sensitivity of key fledging mass and recruitment models to inclusion of father identity as a random effect. Shown are fixed‐effect estimates (on the standardised scale or logit scale, with standard errors) and random‐effect standard deviations for models fitted with and without father identity. Across all model types, including father ID modestly reallocated variance among random effects but did not materially alter the magnitude, direction, or statistical significance of climatic, ECE, clutch‐size, or lay‐date effects.
**Table S9.** Robustness of nestling‐stage ECE effects to potential carry‐over from hatchling exposure. All effects are standardized (SD units). Positive interactions indicate greater nestling‐stage effects for chicks with prior hatchling exposure; negative interactions indicate reduced effects.
**Figure S1.** Path diagram showing standardized regression coefficients (β) for lay date, temperature, ECEs and chickweight relationships. Positive relationships are shown in black, negative relationships in red.
**Table S10.** Path analysis coefficients, standard errors and t‐values.
**Table S11.** Average temperatures during Hot ECE exposure comparing very early broods (≤ 15th percentile relative lay date within each cohort year) and very late (≥ 85th percentile). Hot ECEs defined as daily mean temperature ≥ +4.52°C above monthly mean temperature.
**Table S12.** Average temperatures during Cold ECE exposure comparing very early broods (≤ 15th percentile relative lay date within each cohort year) and very late (≥ 85th percentile). Cold ECEs defined as daily mean temperature ≤ −4.49°C below monthly mean temperature.
**Table S13.** Average rainfall during Rain ECE exposure comparing very early broods (≤ 15th percentile relative lay date within each cohort year) and very late (≥ 85th percentile). Rain ECEs defined as total rainfall in 24 h ≥ 6.20 mm above the monthly mean.
**Table S14A.** Mean temperatures (°C) experienced by broods during both hatchling and nestling periods, grouped by number of ECEs.
**Table S14B.** Mean rainfall (mm) experienced by broods during both hatchling and nestling periods, grouped by number of rain ECEs.
**Table S15A.** Pearson correlations between original (monthly baseline) and brood‐specific ECE counts across developmental stages. Bold values show within‐ECE‐type correlations between methods (original vs. brood‐specific). *n* = 10,892 broods.
**Table S15B.** Linear mixed model parameter estimates for hot ECE effects: original (monthly baseline) versus brood‐specific window methods.
**Figure S2.** Comparison of ECE frequency estimates under the original (monthly baseline) and brood‐specific window methods. Each plot shows distributions of the number of extreme temperature events (ECEs) per brood under the original monthly‐baseline method (pink; ‘Original’) and the brood‐specific ±15‐day window method (blue; ‘Brood‐specific’). Plots show (A) hot ECEs during the hatchling stage, (B) hot ECEs during the nestling stage, (C) cold ECEs during the hatchling stage and (D) cold ECEs during the nestling stage. Reported Pearson correlation coefficients (r) indicate the strength of agreement in brood‐level ECE counts between methods for each stage and ECE type.

## Data Availability

Data and code to reproduce all analyses are available at https://doi.org/10.5281/zenodo.18763378 and in GitHub at https://github.com/devisatarkar/ECEchickweight_great‐tits.
