# Supplementary material for: Developmental Stage‐Specific Responses to Extreme Climatic Events and Environmental Variability in Great Tit Nestlings
Source: Glob Chang Biol. 2026 Mar 11;32(3):e70794. doi: 10.1111/gcb.70794 (PMC12976983; doi:10.1111/gcb.70794)
Supplement: Supplementary file 1 — Table S1. Ambient climate models: outputs of linear mixed models for fledging mass with ambient climate measures (mean temperature/mean rainfall) during specific developmental stages (hatchling/nestling) as explanatory variables along with lay date, and clutch size as fixed effects. Year of birth, brood identity, mother identity and natal nest box are included as random effects. All fixed effects are scaled to a mean of zero and standard deviation of one. Significant terms (p < 0.05) are in bold. Table S1.1. Model selection for ambient temperature effects on fledging mass (based on AICc). Table S2. Extreme climate events (frequency) models: outputs of linear mixed models for fledging mass with number of ECEs during specific developmental stages (hatchling/nestling) as explanatory variables along with mean temperature during the relevant stage, lay date, and clutch size as fixed effects. Year of birth, brood identity, mother identity and natal nest box are included as random effects. All fixed effects are scaled to a mean of zero and standard deviation of one. Significant terms (p < 0.05) are in bold. Here, ECEs are calculated with a 5% threshold. Table S3. Extreme climate events (binary) models: outputs of linear mixed models for fledging mass with presence of at least 1 ECE (1% or 5%) during specific developmental stages (hatchling/nestling) as explanatory variables along with mean temperature during the relevant stage, lay date, and clutch size as fixed effects. Year of birth, brood identity, mother identity and natal nest box are included as random effects. All fixed effects are scaled to a mean of zero and standard deviation of one. Significant terms (p < 0.05) are in bold. Table S4. Interaction models (ambient climate × ECE frequency): outputs of linear mixed models for fledging mass with number of ECEs during specific developmental stages (hatchling/nestling) interacting with relevant ambient climate measures as predictors, along with lay date and clutch size [file GCB-32-e70794-s001.docx]

**Table S1. Ambient climate models:** Outputs of linear mixed models for fledging mass with ambient climate measures (mean temperature/ mean rainfall) during specific developmental stages (hatchling/nestling) as explanatory variables along with laydate, and clutch size as fixed effects. Year of birth, brood identity, mother identity and natal nest box are included as random effects. All fixed effects are scaled to a mean of zero and standard deviation of one. Significant terms (*p <0.05)* are in bold.

| Variable | Estimate | Std. Error | df | t | | p |
| --- | --- | --- | --- | --- | --- | --- |
| *Average temperature (hatchling stage)* | | | | | | |
| Mean temperature | 0.1202 | 0.01994 | 10010 | | 6.03 | **< 0.001** |
| **(Mean temperature)^2^** | **-0.0763** | 0.01099 | 10090 | | -6.94 | **< 0.001** |
| Lay date | -0.3343 | 0.02021 | 10430 | | -16.54 | **< 0.001** |
| Clutch size | -0.2474 | 0.01409 | 10010 | | -17.55 | **< 0.001** |
| *Average temperature (hatchling stage) – using splines (df=5)* | | | | | | |
| **Mean temperature.1** | **0.8245** | 0.1548 | 9863 | | 5.33 | **< 0.001** |
| **Mean temperature.2** | **0.6015** | 0.1795 | 9878 | | 3.35 | **0.0008** |
| **Mean temperature.3** | **0.7902** | 0.1262 | 10060 | | 6.26 | **< 0.001** |
| **Mean temperature.4** | **0.7237** | 0.3826 | 9912 | | 1.89 | **0.0586** |
| Mean temperature.5 | 0.03598 | 0.2200 | 10150 | | 0.16 | 0.8701 |
| Lay date | -0.3317 | 0.02026 | 10400 | | -16.37 | **< 0.001** |
| Clutch size | -0.2477 | 0.01410 | 10020 | | -17.57 | **< 0.001** |
| *Average temperature (nestling stage)* | | | | | | |
| Mean temperature | 0.1344 | 0.01812 | 10340 | | 7.42 | **< 0.001** |
| **(Mean temperature)^2^** | **-0.04692** | 0.01009 | 10130 | | -4.65 | **< 0.001** |
| Lay date | -0.3515 | 0.02039 | 10510 | | -17.24 | **< 0.001** |
| Clutch size | -0.2485 | 0.01412 | 10030 | | -17.61 | **< 0.001** |
| *Average temperature (nestling stage) – using splines (df=5)* | | | | | | |
| **Mean temperature.1** | **0.5032** | 0.1848 | 9981 | | 2.72 | **0.0064** |
| **Mean temperature.2** | **0.3726** | 0.2049 | 9983 | | 1.82 | **0.0690** |
| **Mean temperature.3** | **0.8374** | 0.1546 | 10220 | | 5.42 | **< 0.001** |
| Mean temperature.4 | 0.09307 | 0.4643 | 9937 | | 0.20 | 0.84114 |
| Mean temperature.5 | -0.3220 | 0.4166 | 10070 | | -0.77 | 0.43955 |
| Lay date | -0.3516 | 0.02047 | 10490 | | -17.17 | **< 0.001** |
| Clutch size | -0.2488 | 0.01412 | 10030 | | -17.63 | **< 0.001** |
| *Average rainfall (hatchling stage)* | | | | | | |
| **Mean rainfall** | **-0.06288** | 0.01813 | 10260 | | -3.47 | **0.000526** |
| Lay date | -0.2918 | 0.01871 | 10500 | | -15.59 | **< 0.001** |
| Clutch size | -0.2387 | 0.01408 | 9981 | | -16.96 | **< 0.001** |
| *Average rainfall (nestling stage)* | | | | | | |
| **Mean rainfall** | **-0.1439** | 0.01580 | 10250 | | -9.11 | **< 0.001** |
| Lay date | -0.2857 | 0.01867 | 10480 | | -15.30 | **< 0.001** |
| Clutch size | -0.2349 | 0.01404 | 10000 | | -16.72 | **< 0.001** |

**Table S1.1** Model selection for ambient temperature effects on fledging mass (based on AICc)

| Model (developmental stage) | K | AICc | ΔAICc | AICcWt | Cum.Wt | Res.LL |
| --- | --- | --- | --- | --- | --- | --- |
| Spline (hatchling) | 13 | 249655.4 | 0.0 | 0.99 | 0.99 | -124814.7 |
| Quadratic (hatchling) | 10 | 249664.1 | 8.7 | 0.01 | 1.00 | -124822.1 |
| Spline (nestling) | 13 | 249656.0 | 0.0 | 0.99 | 0.99 | -124815.0 |
| Quadratic (nestling) | 10 | 249665.2 | 9.2 | 0.01 | 1.00 | -124822.6 |

**Table S2. Extreme climate events (frequency) models:** Outputs of linear mixed models for fledging mass with number of ECEs during specific developmental stages (hatchling/nestling) as explanatory variables along with mean temperature during the relevant stage, laydate, and clutch size as fixed effects. Year of birth, brood identity, mother identity and natal nest box are included as random effects. All fixed effects are scaled to a mean of zero and standard deviation of one. Significant terms (*p <0.05)* are in bold. Here, ECEs are calculated with a 5% threshold.

| Variable | Estimate | Std. Error | df | t | p |
| --- | --- | --- | --- | --- | --- |
| *Number of hot ECEs (hatchling stage)* | | | | | |
| Mean temperature | 0.1051 | 0.02749 | 9941 | 3.83 | **< 0.001** |
| Lay date | -0.3332 | 0.02144 | 10350 | -15.54 | **< 0.001** |
| Clutch size | -0.2445 | 0.01416 | 10010 | -17.27 | **< 0.001** |
| Number of hot ECEs | -0.02235 | 0.02258 | 10170 | -0.99 | 0.3222 |
| *Number of hot ECEs (nestling stage)* | | | | | |
| Mean temperature | 0.0614 | 0.0237 | 10320 | 2.59 | **0.009** |
| Lay date | -0.3202 | 0.0216 | 10500 | -14.82 | **< 0.001** |
| Clutch size | -0.2444 | 0.0142 | 10060 | -17.25 | **< 0.001** |
| **Number of hot ECEs** | **0.0914** | 0.0240 | 10180 | 3.81 | **0.00014** |
| *Number of hot ECEs (nestling stage) – using splines* | | | | | |
| Number of hot ECEs.1 | 0.08188 | 0.07484 | 10030 | 1.09 | 0.274 |
| **Number of hot ECEs.2** | **-0.8576** | 0.2392 | 10130 | -3.59 | **< 0.001** |
| **Number of hot ECEs.3** | **1.883** | 0.4019 | 10220 | 4.68 | **< 0.001** |
| Mean temperature | 0.07136 | 0.02424 | 10330 | 2.94 | **0.003** |
| Lay date | -0.3236 | 0.02176 | 10510 | -14.87 | **< 0.001** |
| Clutch size | -0.2447 | 0.01417 | 10050 | -17.27 | **< 0.001** |
| *Number of cold ECEs (hatchling stage)* | | | | | |
| Mean temperature | 0.05067 | 0.02205 | 10170 | 2.30 | **0.0216** |
| Lay date | -0.3224 | 0.02024 | 10400 | -15.93 | **< 0.001** |
| Clutch size | -0.2428 | 0.01411 | 10010 | -17.22 | **< 0.001** |
| **Number of cold ECEs** | **-0.06948** | 0.02089 | 10160 | -3.33 | **0.00089** |
| *Number of cold ECEs (nestling stage)* | | | | | |
| Mean temperature | 0.1734 | 0.02080 | 10210 | 8.34 | **< 0.001** |
| Lay date | -0.3705 | 0.02088 | 10500 | -17.74 | **< 0.001** |
| Clutch size | -0.2524 | 0.01414 | 10050 | -17.85 | **< 0.001** |
| Number of cold ECEs | 0.09059 | 0.01833 | 9961 | 4.97 | **< 0.001** |
| *Number of rain ECEs (hatchling stage)* | | | | | |
| Mean temperature | 0.08192 | 0.01954 | 10070 | 4.19 | **< 0.001** |
| Lay date | -0.3243 | 0.02026 | 10400 | -16.01 | **< 0.001** |
| Clutch size | -0.2431 | 0.01411 | 10010 | -17.23 | **< 0.001** |
| Number of rain ECEs | -0.02431 | 0.01637 | 10320 | -1.49 | 0.137 |
| *Number of rain ECEs (nestling stage)* | | | | | |
| Mean temperature | 0.1095 | 0.01805 | 10270 | 6.06 | **< 0.001** |
| Lay date | -0.3367 | 0.02051 | 10400 | -16.42 | **< 0.001** |
| Clutch size | -0.2451 | 0.01414 | 10030 | -17.33 | **< 0.001** |
| **Number of rain ECEs** | **-0.07346** | 0.01619 | 10270 | -4.54 | **< 0.001** |

**Table S3. Extreme climate events (binary) models:** Outputs of linear mixed models for fledging mass with presence of at least 1 ECE (1% or 5%) during specific developmental stages (hatchling/nestling) as explanatory variables along with mean temperature during the relevant stage, laydate, and clutch size as fixed effects. Year of birth, brood identity, mother identity and natal nest box are included as random effects. All fixed effects are scaled to a mean of zero and standard deviation of one. Significant terms (*p <0.05)* are in bold.

| **Variable** | **β** | **Std. Error** | **df** | **t** | **p** | **β** | **Std. Error** | **df** | **t** | **p** |
| --- | --- | --- | --- | --- | --- | --- | --- | --- | --- | --- |
| *Presence of at least 1 hot (1%) ECE (hatchling)* | | | | | | *Presence of at least 1 hot (5%) ECE (hatchling)* | | | | |
| Presence of hot ECE | -0.014 | 0.018 | 10220 | -0.797 | 0.425 | 0.047 | 0.02 | 10270 | 2.319 | 0.0204 |
| Mean temperature | 0.095 | 0.023 | 9981 | 4.221 | **< 0.001** | 0.048 | 0.025 | 10120 | 1.866 | 0.0621 |
| Clutch size | -0.244 | 0.014 | 10010 | -17.26 | **< 0.001** | -0.241 | 0.014 | 10010 | -17.089 | **< 0.001** |
| Lay date | -0.329 | 0.02 | 10370 | -16.054 | **< 0.001** | -0.314 | 0.021 | 10420 | -14.98 | **< 0.001** |
| *Presence of at least 1 hot (1%) ECE (nestling)* | | | | | | *Presence of at least 1 hot (5%) ECE (nestling)* | | | | |
| Presence of hot ECE | 0.023 | 0.02 | 10210 | 1.138 | 0.255 | 0.012 | 0.02 | 10170 | 0.597 | 0.551 |
| Mean temperature | 0.11 | 0.02 | 10340 | 5.479 | **< 0.001** | 0.113 | 0.022 | 10370 | 5.18 | **< 0.001** |
| Clutch size | -0.248 | 0.014 | 10040 | -17.533 | **< 0.001** | -0.248 | 0.014 | 10040 | -17.547 | **< 0.001** |
| Lay date | -0.343 | 0.021 | 10480 | -16.539 | **< 0.001** | -0.344 | 0.021 | 10520 | -16.164 | **< 0.001** |
| *Presence of at least 1 cold (1%) ECE (hatchling)* | | | | | | *Presence of at least 1 cold (5%) ECE (hatchling)* | | | | |
| Presence of cold ECE | 0.013 | 0.013 | 10240 | 1.004 | 0.315 | -0.031 | 0.02 | 10270 | -1.582 | 0.113785 |
| Mean temperature | 0.088 | 0.019 | 10070 | 4.504 | **< 0.001** | 0.071 | 0.021 | 10100 | 3.339 | **< 0.001** |
| Clutch size | -0.244 | 0.014 | 10010 | -17.259 | **< 0.001** | -0.243 | 0.014 | 10010 | -17.239 | **< 0.001** |
| Lay date | -0.328 | 0.02 | 10400 | -16.158 | **< 0.001** | -0.324 | 0.02 | 10400 | -15.982 | **< 0.001** |
| *Presence of at least 1 cold (1%) ECE (nestling)* | | | | | | *Presence of at least 1 cold (5%) ECE (nestling)* | | | | |
| Presence of cold ECE | 0.087 | 0.017 | 9390 | 4.983 | **< 0.001** | 0.096 | 0.017 | 10280 | 5.667 | **< 0.001** |
| Mean temperature | 0.132 | 0.018 | 10330 | 7.333 | **< 0.001** | 0.174 | 0.02 | 10310 | 8.614 | **< 0.001** |
| Clutch size | -0.249 | 0.014 | 10030 | -17.662 | **< 0.001** | -0.251 | 0.014 | 10050 | -17.776 | **< 0.001** |
| Lay date | -0.356 | 0.02 | 10500 | -17.416 | **< 0.001** | -0.369 | 0.021 | 10520 | -17.817 | **< 0.001** |
| *Presence of at least 1 rain (1%) ECE (hatchling)* | | | | | | *Presence of at least 1 rain (5%) ECE (hatchling)* | | | | |
| Presence of rain ECE | -0.041 | 0.016 | 10160 | -2.578 | **0.00995** | -0.012 | 0.016 | 10240 | -0.751 | 0.453 |
| Mean temperature | 0.084 | 0.019 | 10070 | 4.324 | **< 0.001** | 0.084 | 0.02 | 10080 | 4.293 | **< 0.001** |
| Clutch size | -0.243 | 0.014 | 10000 | -17.235 | **< 0.001** | -0.243 | 0.014 | 10000 | -17.242 | **< 0.001** |
| Lay date | -0.325 | 0.02 | 10410 | -16.065 | **< 0.001** | -0.325 | 0.02 | 10400 | -16.058 | **< 0.001** |
| *Presence of at least 1 rain (1%) ECE (nestling)* | | | | | | *Presence of at least 1 rain (5%) ECE (nestling)* | | | | |
| Presence of rain ECE | -0.042 | 0.017 | 10200 | -2.488 | 0.0129 | -0.077 | 0.016 | 10270 | -4.86 | **< 0.001** |
| Mean temperature | 0.115 | 0.018 | 10300 | 6.364 | **< 0.001** | 0.105 | 0.018 | 10270 | 5.786 | **< 0.001** |
| Clutch size | -0.248 | 0.014 | 10040 | -17.554 | **< 0.001** | -0.246 | 0.014 | 10030 | -17.39 | **< 0.001** |
| Lay date | -0.343 | 0.02 | 10450 | -16.773 | **< 0.001** | -0.341 | 0.02 | 10450 | -16.7 | **< 0.001** |

**Table S4. Interaction models (ambient climate x ECE frequency):** Outputs of linear mixed models for fledging mass with number of ECEs during specific developmental stages (hatchling/nestling) interacting with relevant ambient climate measures as predictors, along with laydate and clutch size as fixed effects. Year of birth, brood identity, mother identity and natal nest box are included as random effects. All fixed effects are scaled to a mean of zero and standard deviation of one. Significant terms (*p <0.05)* are in bold. Here, ECEs are calculated with a 5% threshold.

| Variable | Estimate | Std. Error | df | t | p |
| --- | --- | --- | --- | --- | --- |
| *Average temperature x number of rain ECEs (hatchling stage)* | | | | | |
| Mean temperature | 0.10280 | 0.02055 | 10040 | 5.000 | **< 0.001** |
| (Mean temperature)^2^ | -0.08914 | 0.01140 | 10160 | -7.816 | **< 0.001** |
| Number of rain ECEs | -0.008569 | 0.01932 | 10200 | -0.443 | 0.657 |
| **Number of rain ECEs x mean temp** | **-0.08596** | 0.01729 | 10580 | -4.973 | **< 0.001** |
| Number of rain ECEs x (mean temp**)^2^** | -0.01853 | 0.01240 | 10370 | -1.494 | 0.135 |
| Lay date | -0.32330 | 0.02031 | 10360 | -15.915 | **< 0.001** |
| Clutch size | -0.24490 | 0.01408 | 10020 | -17.391 | **< 0.001** |
| *Average temperature x number of rain ECEs (nestling stage)* | | | | | |
| Mean temperature | 0.12780 | 0.01871 | 10300 | 6.831 | **< 0.001** |
| (Mean temperature)^2^ | -0.04408 | 0.01047 | 10160 | -4.210 | **< 0.001** |
| Number of rain ECEs | **-0.09363** | **0.01879** | 10290 | -4.983 | **< 0.001** |
| Number of rain ECEs x mean temp | -0.01330 | 0.01887 | 10240 | -0.705 | 0.4808 |
| Number of rain ECEs x (mean temp**)^2^** | 0.02973 | 0.01329 | 10150 | 2.236 | 0.0253 |
| Lay date | -0.33750 | 0.02056 | 10410 | -16.418 | **< 0.001** |
| Clutch size | -0.24470 | 0.01413 | 10020 | -17.317 | **< 0.001** |
| *Average rainfall x number of hot ECEs (hatchling stage)* | | | | | |
| Mean rainfall | -0.08781 | 0.01867 | 10280 | -4.703 | **< 0.001** |
| Number of hot ECEs | **-0.06939** | 0.02115 | 10340 | -3.282 | **0.00104** |
| **Number of hot ECEs x mean rainfall** | **-0.16190** | 0.02197 | 10230 | -7.367 | **< 0.001** |
| Lay date | -0.28250 | 0.01872 | 10490 | -15.088 | **< 0.001** |
| Clutch size | 0.23510 | 0.01405 | 9984 | -16.729 | **< 0.001** |
| *Average rainfall x number of hot ECEs (nestling stage)* | | | | | |
| Mean rainfall | -0.11430 | 0.01682 | 10170 | -6.795 | **< 0.001** |
| Number of hot ECEs | **0.12860** | 0.02045 | 9950 | 6.288 | **< 0.001** |
| Number of hot ECEs x mean rainfall | 0.05163 | 0.02041 | 10120 | 2.530 | 0.0114 |
| Lay date | -0.28690 | 0.01864 | 10500 | -15.389 | **< 0.001** |
| Clutch size | -0.23560 | 0.01403 | 10010 | -16.800 | **< 0.001** |
| *Average rainfall x number of cold ECEs (hatchling stage)* | | | | | |
| Mean rainfall | -0.05072 | 0.01831 | 10240 | -2.770 | **0.00561** |
| Number of cold ECEs | **-0.08035** | 0.01874 | 10080 | -4.287 | **< 0.001** |
| Number of cold ECEs x mean rainfall | 0.03919 | 0.01985 | 10170 | 1.974 | 0.04838 |
| Lay date | -0.30360 | 0.01890 | 10510 | -16.070 | **< 0.001** |
| Clutch size | -0.24000 | 0.01407 | 9986 | -17.059 | **< 0.001** |
| *Average rainfall x number of cold ECEs (nestling stage)* | | | | | |
| Mean rainfall | -0.14640 | 0.01593 | 10200 | -9.190 | **< 0.001** |
| Number of cold ECEs | 0.03267 | 0.01587 | 9979 | 2.059 | 0.03952 |
| Number of cold ECEs x mean rainfall | 0.05829 | 0.01624 | 10040 | 3.589 | **0.000334** |
| Lay date | -0.28960 | 0.01870 | 10470 | -15.488 | **< 0.001** |
| Clutch size | -0.23540 | 0.01404 | 10010 | -16.767 | **< 0.001** |

**Table S5. Interaction models (relative laydate x ECE frequency):** Outputs of linear mixed models for fledging mass with number of ECEs during specific developmental stages (hatchling/nestling) interacting with relative lay date as predictors, along with mean temperature and clutch size as fixed effects. Year of birth, brood identity, mother identity and natal nest box are included as random effects. All fixed effects are scaled to a mean of zero and standard deviation of one. Significant terms (*p <0.05)* are in bold. Here, ECEs are calculated with a 5% threshold.

| Variable | Estimate | Std. Error | df | t | p |
| --- | --- | --- | --- | --- | --- |
| *Base model for relative laydate* | | | | | |
| Relative laydate | -0.13960 | 0.01443 | 10010 | -9.676 | **< 0.001** |
| **(Relative laydate)^2^** | **-0.04080** | 0.00464 | 10890 | -8.791 | **< 0.001** |
| Clutch size | -0.23560 | 0.01404 | 9973 | -16.787 | **< 0.001** |
| *Relative laydate x number of hot ECEs (hatchling stage)* | | | | | |
| Relative laydate | -0.16190 | 0.01596 | 10250 | -10.144 | **< 0.001** |
| (Relative laydate)^2^ | -0.04306 | 0.00465 | 10860 | -9.268 | **< 0.001** |
| Mean temperature | 0.11280 | 0.02724 | 9227 | 4.142 | **< 0.001** |
| Number of hot ECEs | -0.02641 | 0.02388 | 9812 | -1.106 | 0.269 |
| **Number of hot ECEs x relative laydate** | **-0.06710** | 0.01295 | 10070 | -5.181 | **< 0.001** |
| Number of hot ECEs x (relative laydate)^2^ | 0.006421 | 0.00493 | 9379 | 1.304 | 0.192 |
| Clutch size | -0.23970 | 0.01409 | 9983 | -17.008 | **< 0.001** |
| *Relative laydate x number of hot ECEs (nestling stage)* | | | | | |
| Relative laydate | -0.15770 | 0.01646 | 10420 | -9.586 | **< 0.001** |
| (Relative laydate)^2^ | -0.03956 | 0.00467 | 10900 | -8.480 | **< 0.001** |
| Mean temperature | 0.05694 | 0.02365 | 10070 | 2.407 | 0.01609 |
| Number of hot ECEs | 0.07277 | 0.02432 | 9859 | 2.992 | **0.00278** |
| Number of hot ECEs x relative laydate | 0.01174 | 0.01738 | 10020 | 0.676 | 0.49926 |
| Number of hot ECEs x (relative laydate)^2^ | 0.00822 | 0.00490 | 9755 | 1.677 | 0.09364 |
| Clutch size | -0.24110 | 0.01414 | 10030 | -17.049 | **< 0.001** |
| *Relative laydate x number of cold ECEs (hatchling stage)* | | | | | |
| Relative laydate | -0.1575 | 0.01522 | 10170 | -10.349 | **< 0.001** |
| (Relative laydate)^2^ | -0.04095 | 0.00501 | 10930 | -8.173 | **< 0.001** |
| Mean temperature | 0.07051 | 0.02191 | 9871 | 3.218 | **0.0013** |
| Number of cold ECEs | -0.01598 | 0.02347 | 9912 | -0.681 | 0.4960 |
| Number of cold ECEs x relative laydate | **0.1000** | 0.01585 | 10420 | 6.313 | **< 0.001** |
| Number of cold ECEs x (relative laydate)^2^ | -0.01439 | 0.00772 | 10770 | -1.864 | 0.0623 |
| Clutch size | -0.2368 | 0.01404 | 9998 | -16.861 | **< 0.001** |
| *Relative laydate x number of cold ECEs (nestling stage)* | | | | | |
| Relative laydate | -0.1964 | 0.01594 | 10410 | -12.321 | **< 0.001** |
| (Relative laydate)^2^ | -0.0441 | 0.00497 | 10890 | -8.881 | **< 0.001** |
| Mean temperature | 0.1621 | 0.02069 | 9708 | 7.834 | **< 0.001** |
| Number of cold ECEs | 0.1432 | 0.02270 | 9561 | 6.305 | **< 0.001** |
| Number of cold ECEs x relative laydate | **0.0377** | 0.01279 | 10040 | 2.945 | **0.00324** |
| Number of cold ECEs x (relative laydate)^2^ | **-0.0394** | 0.00885 | 10680 | -4.449 | **< 0.001** |
| Clutch size | -0.2491 | 0.01409 | 10040 | -17.682 | **< 0.001** |
| *Relative laydate x number of rain ECEs (hatchling stage)* | | | | | |
| Relative laydate | -0.1643 | 0.01522 | 10170 | -10.796 | **< 0.001** |
| (Relative laydate)^2^ | -0.04215 | 0.00464 | 10900 | -9.076 | **< 0.001** |
| Mean temperature | 0.09277 | 0.01939 | 9775 | 4.784 | **< 0.001** |
| Number of rain ECEs | 0.00335 | 0.01818 | 10270 | 0.184 | 0.854 |
| Number of rain ECEs x relative laydate | 0.06374 | 0.01318 | 10360 | 4.835 | **< 0.001** |
| Number of rain ECEs x (relative laydate)^2^ | -0.01935 | 0.00481 | 10600 | -4.020 | **< 0.001** |
| Clutch size | -0.2414 | 0.01404 | 9990 | -17.193 | **< 0.001** |
| *Relative laydate x number of rain ECEs (nestling stage)* | | | | | |
| Relative laydate | -0.1650 | 0.01582 | 10350 | -10.433 | **< 0.001** |
| (Relative laydate)^2^ | -0.04319 | 0.00479 | 10930 | -9.024 | **< 0.001** |
| Mean temperature | 0.09695 | 0.01805 | 10060 | 5.372 | **< 0.001** |
| Number of rain ECEs | **-0.05820** | 0.01751 | 10160 | -3.323 | **0.000893** |
| Number of rain ECEs x relative laydate | -0.01501 | 0.01628 | 10190 | -0.922 | 0.356464 |
| **Number of rain ECEs x (relative laydate)^2^** | **-0.01414** | 0.00606 | 10550 | -2.334 | **0.019614** |
| Clutch size | -0.2411 | 0.01410 | 10010 | -17.100 | **< 0.001** |

**Table S6. Local recruitment models:** Outputs of generalised linear mixed models for recruitment probability with number of ECEs during specific developmental stages (hatchling/nestling) as predictors, along with clutch size as fixed effect. Outputs of additional models with laydate as fixed effect have also been detailed below. ECEs are treated here as categorical variables. Year of birth, brood identity, mother identity and natal nest box are included as random effects. All fixed effects are scaled to a mean of zero and standard deviation of one. Significant terms (*p <0.05)* are in bold. Here, ECEs are calculated with a 5% threshold.

| Variable | β | Std. Error | z | p | β | Std. Error | z | p |
| --- | --- | --- | --- | --- | --- | --- | --- | --- |
| *Number of Cold ECEs (hatchling stage)* | | | | | *with laydate* | | | |
| **1 Cold ECE** | **-0.2418** | 0.07388 | -3.273 | **0.00106** | -0.14016 | 0.07507 | -1.867 | 0.0619 |
| 2 Cold ECEs | -0.02525 | 0.08889 | -0.284 | 0.77642 | -0.03618 | 0.08958 | -0.404 | 0.6863 |
| **3 Cold ECEs** | **-0.26187** | 0.10499 | -2.494 | **0.01262** | **-0.22566** | 0.10568 | -2.135 | **0.0327** |
| **4+ Cold ECEs** | **-0.30369** | 0.12826 | -2.368 | **0.01789** | -0.20996 | 0.13088 | -1.604 | 0.1087 |
| Mean temperature | -0.14797 | 0.02337 | -6.332 | **< 0.001** | -0.03616 | 0.02486 | -1.454 | 0.1459 |
| Clutch size | -0.04323 | 0.01513 | -2.858 | **0.00427** | -0.1079 | 0.01591 | -6.783 | **< 0.001** |
| Lay date | – | – | – | **–** | -0.33045 | 0.02486 | -13.295 | **< 0.001** |
| *Number of Cold ECEs (nestling stage)* | | | | | *with laydate* | | | |
| **1 Cold ECE** | **-0.15801** | 0.06662 | -2.372 | **0.017695** | -0.02927 | 0.0683 | -0.429 | 0.6683 |
| 2 Cold ECEs | 0.041865 | 0.13519 | 0.31 | 0.756821 | 0.1619 | 0.13507 | 1.199 | 0.2307 |
| 3 Cold ECEs | 0.004844 | 0.12191 | 0.04 | 0.968308 | 0.19524 | 0.12305 | 1.587 | 0.1126 |
| **4+ Cold ECEs** | **-0.56687** | 0.15759 | -3.597 | **0.000322** | -0.16141 | 0.16179 | -0.998 | 0.3184 |
| Mean temperature | -0.17697 | 0.02122 | -8.337 | **< 0.001** | -0.04842 | 0.02408 | -2.011 | **0.0444** |
| Clutch size | -0.04353 | 0.01513 | -2.878 | **0.004008** | -0.10503 | 0.01599 | -6.569 | **< 0.001** |
| Lay date | – | – | – | **–** | -0.31035 | 0.0259 | -11.984 | **< 0.001** |
| *Number of Rain ECEs (hatchling stage)* | | | | | *with laydate* | | | |
| 1 Rain ECE | -0.04466 | 0.04182 | -1.068 | 0.2856 | -0.005418 | 0.042032 | -0.129 | 0.897 |
| 2 Rain ECEs | -0.07559 | 0.06781 | -1.115 | 0.2649 | -0.035606 | 0.068786 | -0.518 | 0.605 |
| 3+ Rain ECEs | 0.13694 | 0.19798 | 0.692 | 0.4891 | 0.120993 | 0.205366 | 0.589 | 0.556 |
| Mean temperature | -0.12121 | 0.02063 | -5.874 | **< 0.001** | -0.011484 | 0.022206 | -0.517 | 0.605 |
| Clutch size | -0.04333 | 0.01513 | -2.863 | **0.0042** | -0.110118 | 0.015911 | -6.921 | **< 0.001** |
| Lay date | – | – | – | **–** | -0.33384 | 0.024779 | -13.473 | **< 0.001** |
| *Number of Rain ECEs (nestling stage)* | | | | | *with laydate* | | | |
| 1 Rain ECE | -0.002987 | 0.042626 | -0.07 | 0.9441 | -0.016176 | 0.042829 | -0.378 | 0.7057 |
| 2 Rain ECEs | -0.079729 | 0.062284 | -1.28 | 0.2005 | -0.001325 | 0.063122 | -0.021 | 0.9833 |
| **3+ Rain ECEs** | **-0.275035** | 0.128208 | -2.145 | **0.0319** | -0.114075 | 0.129947 | -0.878 | 0.38 |
| Mean temperature | -0.149838 | 0.01889 | -7.932 | **< 0.001** | -0.051995 | 0.020788 | -2.501 | **0.0124** |
| Clutch size | -0.040311 | 0.015127 | -2.665 | **0.0077** | -0.105978 | 0.015981 | -6.632 | **< 0.001** |
| Lay date | – | – | – | **–** | -0.313345 | 0.025328 | -12.371 | **< 0.001** |
| *Number of Hot ECEs (hatchling stage)* | | | | | *with laydate* | | | |
| **1 Hot ECE** | **0.12491** | 0.0626 | 1.995 | **0.046018** | -0.022391 | 0.064208 | -0.349 | 0.727 |
| **2 Hot ECEs** | **0.34596** | 0.0948 | 3.649 | **0.000263** | 0.031739 | 0.098806 | 0.321 | 0.748 |
| **3 Hot ECEs** | **0.24191** | 0.10574 | 2.288 | **0.022148** | -0.164768 | 0.111179 | -1.482 | 0.138 |
| **4 Hot ECEs** | **0.46152** | 0.1761 | 2.621 | **0.008771** | 0.088114 | 0.180568 | 0.488 | 0.626 |
| **5+ Hot ECEs** | **0.52201** | 0.15026 | 3.474 | **0.000513** | 0.029637 | 0.156089 | 0.19 | 0.849 |
| Mean temperature | -0.19485 | 0.02826 | -6.895 | **< 0.001** | -0.001021 | 0.032092 | -0.032 | 0.975 |
| Clutch size | -0.04543 | 0.01514 | -3.002 | **0.002686** | -0.111208 | 0.015944 | -6.975 | **< 0.001** |
| Lay date | – | – | – | **–** | -0.338837 | 0.025942 | -13.061 | **< 0.001** |
| *Number of Hot ECEs (nestling stage)* | | | | | *with laydate* | | | |
| **1 Hot ECE** | **0.23385** | 0.05376 | 4.35 | **< 0.001** | 0.08144 | 0.05566 | 1.463 | 0.143399 |
| **2 Hot ECEs** | **0.34338** | 0.08938 | 3.842 | **0.000122** | 0.1952 | 0.09084 | 2.149 | **0.031642** |
| **3 Hot ECEs** | **0.52889** | 0.10655 | 4.964 | **< 0.001** | 0.23387 | 0.11032 | 2.12 | **0.034011** |
| **4 Hot ECEs** | **0.55928** | 0.11811 | 4.735 | **< 0.001** | 0.23986 | 0.12172 | 1.971 | **0.048772** |
| **5+ Hot ECEs** | **0.7122** | 0.12106 | 5.883 | **< 0.001** | 0.27359 | 0.12834 | 2.132 | **0.033026** |
| Mean temperature | -0.25472 | 0.02409 | -10.572 | **< 0.001** | -0.09981 | 0.02776 | -3.595 | **0.000324** |
| Clutch size | -0.04643 | 0.01515 | -3.065 | **0.002174** | -0.10275 | 0.01602 | -6.416 | **< 0.001** |
| Lay date | – | – | – | **–** | -0.29293 | 0.02654 | -11.037 | **< 0.001** |

**Table S7.** Higher frequencies of ECEs were grouped into a single categorical level, due to the reduced number of individuals experiencing high frequencies of ECEs. Local recruitment models from Table 6 used categorical ECE variables for analysis. Blue shaded areas indicate the values that were combined for each type of ECE in each developmental period.

| **ECE** **type** | **Frequency** | | | | | | | |
| --- | --- | --- | --- | --- | --- | --- | --- | --- |
|  | 0 | 1 | 2 | 3 | 4 | 5 | 6 | 7 |
| **Hatchling** |  |  |  |  |  |  |  |  |
| Hot | 68167 | 8084 | 2568 | 3262 | 595 | 633 | 565 | 61 |
| Cold | 70622 | 5036 | 4015 | 2382 | 1443 | 424 | 13 | 0 |
| Rain | 59522 | 17499 | 6432 | 459 | 23 | 0 | 0 | 0 |
| **Nestling** |  |  |  |  |  |  |  |  |
| Hot | 60344 | 12561 | 3821 | 2537 | 2055 | 1594 | 934 | 84 |
| Cold | 74884 | 5902 | 994 | 1153 | 440 | 463 | 99 | 0 |
| Rain | 59181 | 16132 | 6890 | 1495 | 237 | 0 | 0 | 0 |

**Table S8.** Sensitivity of key fledging mass and recruitment models to inclusion of father identity as a random effect. Shown are fixed-effect estimates (on the standardised scale or logit scale, with standard errors) and random-effect standard deviations for models fitted with and without father identity. Across all model types, including father ID modestly reallocated variance among random effects but did not materially alter the magnitude, direction, or statistical significance of climatic, ECE, clutch-size, or lay-date effects.

| *Average temperature (hatchling stage) - FLEDGING MASS* | | |
| --- | --- | --- |
| *Fixed effects* | Estimate (SE) – no father ID | Estimate (SE) – with father ID |
| Mean temperature | 0.120 (0.019) | 0.0127 (0.019) |
| (Mean temperature)^2^ | -0.076 (0.01) | -0.071 (0.01) |
| Lay date | -0.334 (0.02) | -0.321 (0.02) |
| Clutch size | -0.247 (0.01) | -0.258 (0.01) |
| *Random effects* | Variance (SD) | Variance (SD) |
| Brood ID | 1.180 (1.08) | 0.869 (0.93) |
| nestbox | 0.072 (0.27) | 0.064 (0.25) |
| birthyear | 0.724 (0.85) | 0.663 (0.81) |
| Mother ID | 0.252 (0.5) | 0.210 (0.45) |
| Father ID | – | 0.131 (0.36) |
| Residual | 0.975 (0.98) | 0.947 (0.97) |
| *Average temperature (hatchling stage) – splines - FLEDGING MASS* | | |
| *Fixed effects* | Estimate (SE) – no father ID | Estimate (SE) – with father ID |
| Mean temperature.1 | 0.824 (0.15) | 0.814 (0.14) |
| Mean temperature.2 | 0.601 (0.17) | 0.571 (0.17) |
| Mean temperature.3 | 0.790 (0.12) | 0.732 (0.12) |
| Mean temperature.4 | 0.723 (0.38) | 0.680 (0.36) |
| Mean temperature.5 | 0.0359 (0.22) | 0.284 (0.21) |
| Lay date | -0.331 (0.02) | -0.319 (0.02) |
| Clutch size | -0.247 (0.01) | -0.259 (0.01) |
| *Random effects* | Variance (SD) | Variance (SD) |
| Brood ID | 1.178 (1.08) | 0.865 (0.93) |
| nestbox | 0.072 (0.27) | 0.063 (0.25) |
| birthyear | 0.717 (0.84) | 0.657 (0.81) |
| Mother ID | 0.253 (0.5) | 0.212 (0.46) |
| Father ID | – | 0.131 (0.36) |
| Residual | 0.975 (0.98) | 0.947 (0.97) |
| *Average rainfall (nestling stage) - FLEDGING MASS* | | |
| *Fixed effects* | Estimate (SE) – no father ID | Estimate (SE) – with father ID |
| Mean rainfall | -0.143 (0.015) | -0.149 (0.015) |
| Lay date | -0.285 (0.018) | -0.271 (0.018) |
| Clutch size | -0.234 (0.014) | -0.245 (0.014) |
| *Random effects* | Variance (SD) | Variance (SD) |
| Brood ID | 1.178 (1.08) | 0.869 (0.93) |
| nestbox | 0.072 (0.27) | 0.064 (0.25) |
| birthyear | 0.684 (0.82) | 0.624 (0.79) |
| Mother ID | 0.252 (0.5) | 0.212 (0.46) |
| Father ID | – | 0.127 (0.36) |
| Residual | 0.975 (0.98) | 0.947 (0.97) |
| *Number of hot ECEs (nestling stage) - FLEDGING MASS* | | |
| *Fixed effects* | Estimate (SE) – no father ID | Estimate (SE) – with father ID |
| No. of hot ECEs | 0.091 (0.02) | 0.07 (0.02) |
| Mean temperature | 0.061 (0.02) | 0.08 (0.02) |
| Lay date | -0.32 (0.02) | -0.31 (0.02) |
| Clutch size | -0.24 (0.01) | -0.25 (0.01) |
| *Random effects* | Variance (SD) | Variance (SD) |
| Brood ID | 1.181 (1.08) | 0.87 (0.93) |
| nestbox | 0.072 (0.27) | 0.065 (0.25) |
| birthyear | 0.744 (0.86) | 0.68 (0.82) |
| Mother ID | 0.252 (0.5) | 0.210 (0.45) |
| Father ID | – | 0.129 (0.36) |
| Residual | 0.975 (0.98) | 0.947 (0.97) |
| *Number of cold ECEs (hatchling stage) - FLEDGING MASS* | | |
| *Fixed effects* | Estimate (SE) – no father ID | Estimate (SE) – with father ID |
| No. of cold ECEs | -0.07 (0.02) | -0.06 (0.02) |
| Mean temperature | 0.05 (0.02) | 0.063 (0.02) |
| Lay date | -0.32 (0.02) | -0.31 (0.02) |
| Clutch size | -0.24 (0.01) | -0.25 (0.01) |
| *Random effects* | Variance (SD) | Variance (SD) |
| Brood ID | 1.184 (1.08) | 0.87 (0.93) |
| nestbox | 0.072 (0.27) | 0.063 (0.25) |
| birthyear | 0.717 (0.86) | 0.65 (0.82) |
| Mother ID | 0.255 (0.5) | 0.211 (0.45) |
| Father ID | – | 0.131 (0.36) |
| Residual | 0.975 (0.98) | 0.947 (0.97) |
| *Average temperature x number of rain ECEs (hatchling stage) - FLEDGING MASS* | | |
| *Fixed effects* | Estimate (SE) – no father ID | Estimate (SE) – with father ID |
| Mean temp | 0.103 (0.021) | 0.105 (0.020) |
| Mean temp² | –0.089 (0.011) | –0.086 (0.011) |
| No of Rain ECEs | –0.009 (0.019) | –0.014 (0.019) |
| Temp × rain ECE | –0.086 (0.017) | –0.081 (0.017) |
| Temp² × rain ECE | –0.019 (0.012) | –0.026 (0.012) |
| Lay date | –0.323 (0.020) | –0.310 (0.020) |
| Clutch size | –0.245 (0.014) | –0.256 (0.014) |
| *Random effects* | Variance (SD) | Variance (SD) |
| Brood ID | 1.177 (1.08) | 0.87 (0.93) |
| nestbox | 0.072 (0.27) | 0.063 (0.25) |
| birthyear | 0.701 (0.83) | 0.64 (0.79) |
| Mother ID | 0.252 (0.5) | 0.209 (0.45) |
| Father ID | – | 0.134 (0.36) |
| Residual | 0.975 (0.98) | 0.947 (0.97) |
| *Number of cold ECEs (hatchling stage) - LOCAL RECRUITMENT* | | |
| *Fixed effects* | Estimate (SE) – no father ID | Estimate (SE) – with father ID |
| Mean temperature | –0.036 (0.025) | –0.032 (0.026) |
| 1 cold day | –0.140 (0.075) | –0.132 (0.078) |
| 2 cold days | –0.036 (0.090) | –0.012 (0.093) |
| 3 cold days | –0.226 (0.106) | –0.214 (0.108) |
| 4+ cold days | –0.210 (0.131) | –0.197 (0.134) |
| Clutch size | –0.108 (0.016) | –0.118 (0.017) |
| Lay date | –0.330 (0.025) | –0.319 (0.026) |
| *Random effects* | Variance (SD) | Variance (SD) |
| Brood ID | 0.208 (0.45) | 0.138 (0.37) |
| nestbox | 0.023 (0.15) | 0.025 (0.16) |
| birthyear | 0.431 (0.65) | 0.414 (0.64) |
| Mother ID | 0.06 (0.24) | 0.036 (0.19) |
| Father ID | – | 0.083 (0.28) |

**Table S9.** Robustness of nestling-stage ECE effects to potential carry-over from hatchling exposure.  All effects are standardized (SD units). Positive interactions indicate greater nestling-stage effects for chicks with prior hatchling exposure; negative interactions indicate reduced effects.

| **ECE Type** | **Analysis** | **N** | **Estimate (SE)** | **df** | **t-value** | **p-value** |
| --- | --- | --- | --- | --- | --- | --- |
| Cold | Full dataset | 83,935 | 0.091 (0.018) | 9961 | 4.94 | <0.001 |
|  | No hatchling ECEs | 70,622 | 0.086 (0.022) | 7527 | 3.88 | <0.001 |
|  | Hatchling ECE x Nestling ECE | 83,935 | -0.024 (0.015) | 10140 | -1.62 | 0.106 |
| Rain | Full dataset | 83,935 | -0.074 (0.016) | 10270 | -4.54 | <0.001 |
|  | No hatchling ECEs | 59,522 | -0.089 (0.022) | 7009 | -4.09 | <0.001 |
|  | Hatchling ECE x Nestling ECE | 83,935 | -0.039 (0.016) | 10300 | -2.38 | 0.017 |
| Hot | Full dataset | 83,935 | 0.091 (0.024) | 10180 | 3.81 | <0.001 |
|  | No hatchling ECEs | 68,167 | 0.062 (0.028) | 8091 | 2.22 | 0.027 |
|  | Hatchling ECE x Nestling ECE | 83,935 | 0.043 (0.015) | 9984 | 2.86 | 0.004 |

**Path analysis for disentangling the effects of laydate on fledging mass**

**
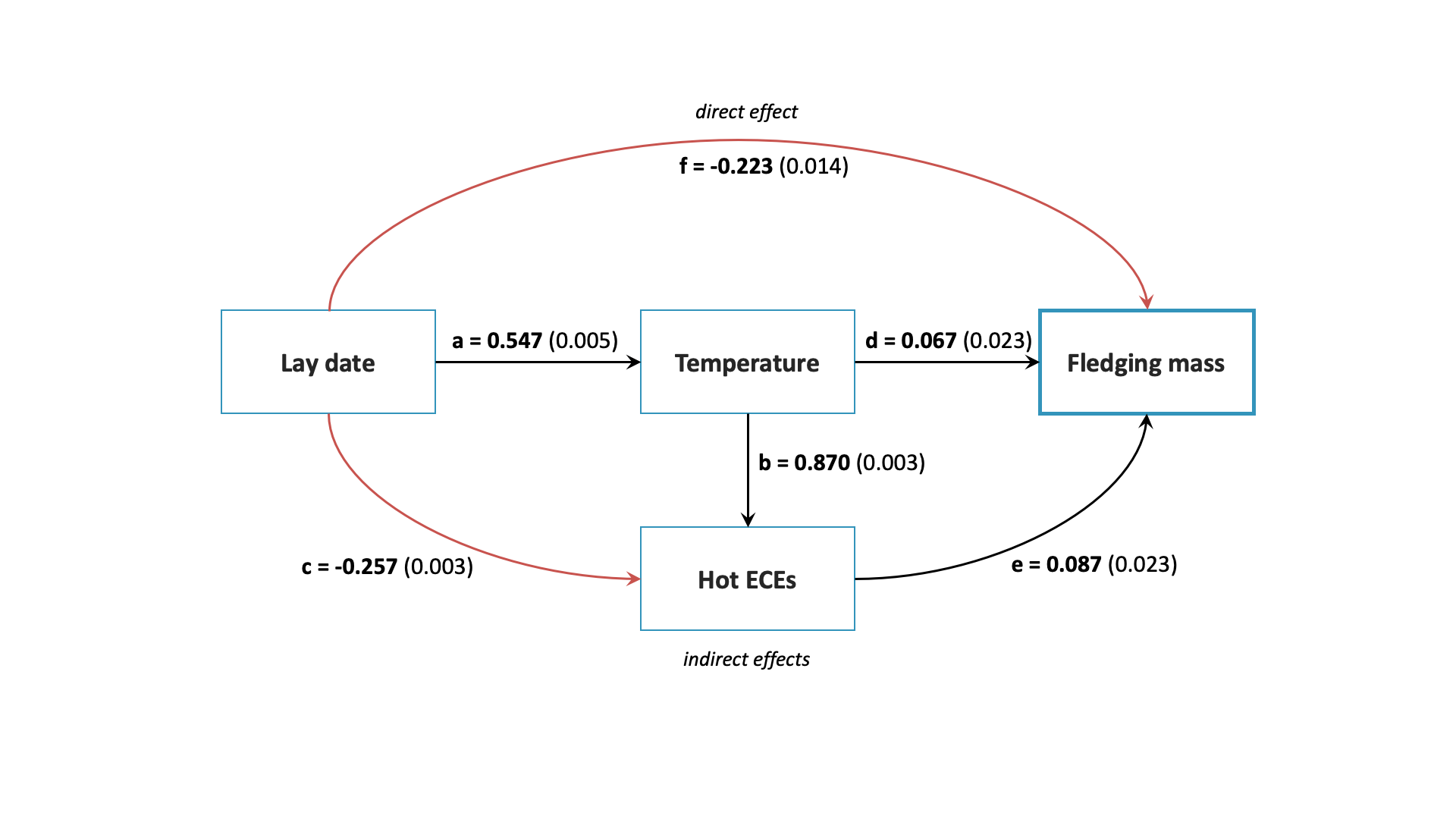
**

**Figure S1.** Path diagram showing standardized regression coefficients (β) for lay date, temperature, ECEs, and chickweight relationships. Positive relationships are shown in black, negative relationships in red.

**Methodology:** We constructed 3 linear mixed models (using lme4) with random effects (RE) of year of birth, brood identity, mother identity and natal nest box. Clutch size was included as a covariate to account for its well-established effect on fledging weight. Indirect effects calculated as products of constituent paths.

Model 1: **Average temperature** ~ **relative laydate** + RE

Model 2: **Number of hot ECEs ~ relative laydate + average temperature**

Model 3: **Fledging mass ~ relative laydate + average temperature + number of hot ECEs** + clutch size + RE

Paths were constructed as follows:

Path a: laydate → temp (model 1) = +0.547

Path b: temp → ECE (model 2) = +0.870

Path c: laydate → ECE (model 2) = -0.257

Path d: temp → chickweight (model 3) = +0.0677

Path e: ECE → chickweight (model 3) = +0.0870

Path f: laydate → chickweight (model 3) = -0.2232

Direct effect of laydate on mass = f = -0.2232

Indirect effects of laydate on mass via temperature and ECEs =

(a * d) + (a * b * e) + (c * e) = (0.547 × 0.0677) + (0.547 x 0.870 x 0.087) + (-0.257 × 0.0870)

Here, (a*d) are laydate effects via temperature only,

(a*b*e) are laydate effects via indirect temperature effects via ECEs,

And (c*e) are laydate effects on mass via ECEs only.

Thus, overall indirect effects of laydate are = 0.0371 - 0.0223 + 0.0414 = 0.0562

Total laydate effects = f + 0.0562 = **-0.167**

And thus, weather-mediated indirect effects are 33.6% of the total laydate effects on fledging mass

**Table S10.** Path analysis coefficients, standard errors, and t-values

| Variable | Estimate | Std. Error | df | t | p | vif |
| --- | --- | --- | --- | --- | --- | --- |
| *Model 1: average temp ~ relative laydate* | | | | | |  |
| Relative laydate | 0.5475 | 0.005 | 6181 | 97.41 | **< 0.001** | – |
| *Model 2: Hot ECEs ~ relative laydate + average temperature* | | | | | |  |
| Relative laydate | -0.257 | 0.003 | – | -75.83 | **< 0.001** | 1.094851 |
| Average temperature | 0.870 | 0.003 | – | 255.39 | **< 0.001** | 1.094851 |
| *Model 3: Fledging mass ~ relative laydate + average temperature + hot ECEs* | | | | | |  |
| Relative laydate | -0.223 | 0.014 | 10840 | -15.397 | **< 0.001** | 1.446125 |
| Average temperature | 0.067 | 0.023 | 10104 | 2.864 | **0.004** | 2.086233 |
| Number of hot ECEs | 0.087 | 0.023 | 9887 | 3.646 | **< 0.001** | 1.753853 |
| Clutch size | -0.246 | 0.014 | 10060 | -17.36 | **< 0.001** | 1.103718 |

**Table S11.** Average temperatures during Hot ECE exposure comparing very early broods (≤15th percentile relative laydate within each cohort year) and very late (≥85th percentile). Hot ECEs defined as daily mean temperature ≥+4.52°C above monthly mean temperature.

| Brood Timing | ECE Count | Mean Temperature (°C) | Number of Broods (n) | Mean Temperature (°C) | Number of Broods (n) |
| --- | --- | --- | --- | --- | --- |
|  |  | *HATCHLING STAGE* | | *NESTLING STAGE* | |
| Early | 0 | 10.8 | 1,413 | 11.4 | 1,386 |
| Early | 1 | 12.5 | 127 | 13.7 | 188 |
| Early | 2 | 13.6 | 88 | 13.6 | 40 |
| Early | 3 | 14.5 | 48 | 15.6 | 67 |
| Early | 4 | 15.6 | 7 | 14.6 | 17 |
| Early | 5 | 16.5 | 12 | 15.5 | 8 |
| Early | 6 | 16.5 | 30 | 16.5 | 17 |
| Late | 0 | 12.5 | 1,332 | 13.3 | 1,446 |
| Late | 1 | 13.9 | 210 | 15.2 | 118 |
| Late | 2 | 13.9 | 80 | 15.8 | 60 |
| Late | 3 | 16.3 | 34 | 17.2 | 30 |
| Late | 4 | 16.2 | 25 | 16.7 | 29 |
| Late | 5 | 15.8 | 22 | 16.8 | 23 |
| Late | 6 | 16.8 | 17 | 17.6 | 11 |

**Table S12.** Average temperatures during Cold ECE exposure comparing very early broods (≤15th percentile relative laydate within each cohort year) and very late (≥85th percentile). Cold ECEs defined as daily mean temperature ≤ -4.49°C below monthly mean temperature.

| Brood Timing | ECE Count | Mean Temperature (°C) | Number of Broods (n) | Mean Temperature (°C) | Number of Broods (n) |
| --- | --- | --- | --- | --- | --- |
|  |  | *HATCHLING STAGE* | | *NESTLING STAGE* | |
| Early | 0 | 11.8 | 1366 | 12.3 | 1517 |
| Early | 1 | 10.7 | 108 | 10.4 | 101 |
| Early | 2 | 9.2 | 115 | 8.8 | 45 |
| Early | 3 | 8.3 | 93 | 9.7 | 43 |
| Early | 4 | 7.6 | 42 | 7.9 | 10 |
| Early | 5 | 7.3 | 1 | 7.2 | 9 |
| Late | 0 | 13.1 | 1521 | 14 | 1527 |
| Late | 1 | 11.9 | 164 | 12.1 | 124 |
| Late | 2 | 10.9 | 15 | 11.3 | 7 |
| Late | 3 | 11.3 | 8 | 11.6 | 30 |
| Late | 4 | 9.4 | 4 | 9.9 | 12 |
| Late | 5 | 9.6 | 8 | 9.7 | 8 |
| Late | 6 | 9.3 | 1 | 9.3 | 13 |

**Table S13.** Average rainfall during Rain ECE exposure comparing very early broods (≤15th percentile relative laydate within each cohort year) and very late (≥85th percentile). Rain ECEs defined as total rainfall in 24 hours ≥ 6.20 mm above the monthly mean.

| Brood Timing | ECE Count | Mean Rainfall (mm) | Number of Broods (n) | Mean Rainfall (mm) | Number of Broods (n) |
| --- | --- | --- | --- | --- | --- |
|  |  | *HATCHLING STAGE* | | *NESTLING STAGE* | |
| Early | 0 | 1 | 1286 | 1.2 | 1225 |
| Early | 1 | 3.1 | 304 | 2.9 | 407 |
| Early | 2 | 4.4 | 107 | 4.6 | 88 |
| Early | 3 | 6 | 28 | 5.2 | 5 |
| Late | 0 | 1 | 1263 | 1.1 | 1264 |
| Late | 1 | 2.9 | 285 | 2.9 | 234 |
| Late | 2 | 4.4 | 151 | 4.6 | 173 |
| Late | 3 | 5.5 | 20 | 6.1 | 36 |
| Late | 4 | 8.4 | 2 | 6.7 | 14 |

**Table S14a.** Mean temperatures (ºC) experienced by broods during both hatchling and nestling periods, grouped by number of ECEs.

| ECE type | ECE Count | Mean Temperature (ºC) | Number of Broods (n) | Mean Temperature (ºC) | Number of Broods (n) |
| --- | --- | --- | --- | --- | --- |
|  |  | *HATCHLING STAGE* | | *NESTLING STAGE* | |
| Hot | 0 | 11.2 | 68167 | 12.2 | 60344 |
| Hot | 1 | 13.5 | 8084 | 13.7 | 12561 |
| Hot | 2 | 13.9 | 2568 | 14.4 | 3821 |
| Hot | 3 | 15.3 | 3262 | 15.9 | 2537 |
| Hot | 4 | 15.2 | 595 | 16.2 | 2055 |
| Hot | 5 | 15.7 | 633 | 16 | 1594 |
| Hot | 6 | 16.5 | 565 | 16.9 | 934 |
| Hot | 7 | 17 | 61 | 17.2 | 84 |
| Cold | 0 | 12.2 | 70622 | 13.1 | 74884 |
| Cold | 1 | 10.8 | 5036 | 12 | 5902 |
| Cold | 2 | 9.2 | 4015 | 10.3 | 994 |
| Cold | 3 | 9.4 | 2382 | 10.6 | 1153 |
| Cold | 4 | 7.9 | 1443 | 9.5 | 440 |
| Cold | 5 | 7.8 | 424 | 9.2 | 463 |
| Cold | 6 | 9.4 | 13 | 9.3 | 99 |

**Table S14b.** Mean rainfall (mm) experienced by broods during both hatchling and nestling periods, grouped by number of rain ECEs.

| ECE type | ECE Count | Mean Rainfall (mm) | Number of Broods (n) | Mean Rainfall (mm) | Number of Broods (n) |
| --- | --- | --- | --- | --- | --- |
|  |  | *HATCHLING STAGE* | | *NESTLING STAGE* | |
| Rain | 0 | 1.1 | 59522 | 1 | 59181 |
| Rain | 1 | 2.9 | 17499 | 2.9 | 16132 |
| Rain | 2 | 5.3 | 6432 | 4.7 | 6890 |
| Rain | 3 | 5.5 | 459 | 5.5 | 1495 |
| Rain | 4 | 7.8 | 23 | 6.8 | 237 |

**Characterising ECEs using brood-specific windows**

**Methodology:** To evaluate the robustness of our ECE metrics, we implemented an alternative, brood-specific approach to calculating temperature ECEs. In contrast to the main analyses, where daily deviations were calculated relative to monthly means across 1965–2024 to obtain fixed 5th/95th percentile thresholds (hot ECE ≥ +4.52°C, cold ECE ≤ −4.49°C), here we defined a local climatic baseline for each brood’s developmental window.

For each brood, we first identified the hatch date (hd, in Julian days), and defined the two developmental periods used in the main analyses: hatchling period (P1: hd to hd+7) and nestling period (P2: hd+8 to hd+15). We then constructed a 31‑day window centred on the midpoint of hd to hd+15 (i.e. ±15 days around hd+7). Using the full climate time series (1965–2024), we calculated the long‑term mean temperature of this 31‑day window across all years. Daily deviations in the focal brood year were then computed as the difference between the observed daily mean temperature and this brood-specific window mean. As in the main analyses, days with deviations ≥ +4.52°C were classified as hot ECEs and ≤ −4.49°C as cold ECEs.

For each brood and developmental stage (P1, P2), we summed the number of hot and cold ECE days under this brood-specific method and compared them to the original monthly-baseline ECE counts. We then re-fitted the linear mixed models for fledging mass using these alternative ECE frequencies to assess whether effect sizes and significance levels were sensitive to the choice of ECE definition.

**Table S15a.** Pearson correlations between original (monthly baseline) and brood-specific ECE counts across developmental stages. Bold values show within-ECE-type correlations between methods (original vs brood-specific). n = 10,892 broods.

|  | **Hot ECE P1** | **Hot ECE P2** | **Cold ECE P1** | **Cold ECE P2** |
| --- | --- | --- | --- | --- |
| **Hot ECE P1** | **0.83** | 0.13 | -0.12 | -0.03 |
| **Hot ECE P2** | 0.13 | **0.90** | 0.15 | -0.08 |
| **Cold ECE P1** | -0.12 | 0.15 | **0.84** | 0.09 |
| **Cold ECE P2** | -0.03 | -0.08 | 0.09 | **0.70** |

**Table S15b.** Linear mixed model parameter estimates for hot ECE effects: Original (monthly baseline) vs brood-specific window methods

| **NESTLING STAGE** | **Original method** | **Brood-specific method** |
| --- | --- | --- |
| **Intercept** | 18.35 (SE = 0.113, t = 162.6, p < 0.001) | 18.35 (SE = 0.112, t = 163.3, p < 0.001) |
| **Mean Temperature** | 0.061 (SE = 0.024, t = 2.59, p = 0.010) | 0.038 (SE = 0.025, t = 1.53, p = 0.126) |
| **Laydate** | -0.320 (SE = 0.022, t = -14.8, p < 0.001) | -0.309 (SE = 0.022, t = -14.2, p < 0.001) |
| **Clutch size** | -0.244 (SE = 0.014, t = -17.2, p < 0.001) | -0.244 (SE = 0.014, t = -17.2, p < 0.001) |
| **Number of Hot ECEs** | **0.091 (SE = 0.024, t = 3.81, p = 0.00014)** | **0.124 (SE = 0.025, t = 4.89, p < 0.0001)** |
| **HATCHLING STAGE** | **Original method** | **Brood-specific method** |
| **Intercept** | 18.35 (SE = 0.111, t = 165.2, p < 0.001) | 18.35 (SE = 0.111, t = 165.4, p < 0.001) |
| **Mean Temperature** | 0.105 (SE = 0.027, t = 3.83, p = 0.00013) | 0.082 (SE = 0.027, t = 3.09, p = 0.0020) |
| **April laydate** | -0.333 (SE = 0.021, t = -15.5, p < 0.001) | -0.325 (SE = 0.022, t = -14.9, p < 0.001) |
| **Clutch size** | -0.245 (SE = 0.014, t = -17.3, p < 0.001) | -0.243 (SE = 0.014, t = -17.2, p < 0.001) |
| **Number of Hot ECEs** | -0.022 (SE = 0.022, t = -0.99, p = 0.322) | 0.004 (SE = 0.021, t = 0.21, p = 0.834) |

Overall, the alternative brood-specific method produced highly correlated ECE counts and closely similar model estimates to the original method (see Table S15A, S15B and Fig. S2), indicating that our main conclusions are robust to the choice of climatic baseline. Distributions overlap strongly in all cases (r = 0.70–0.90), indicating close agreement between the two ECE calculation methods (Fig. S2).


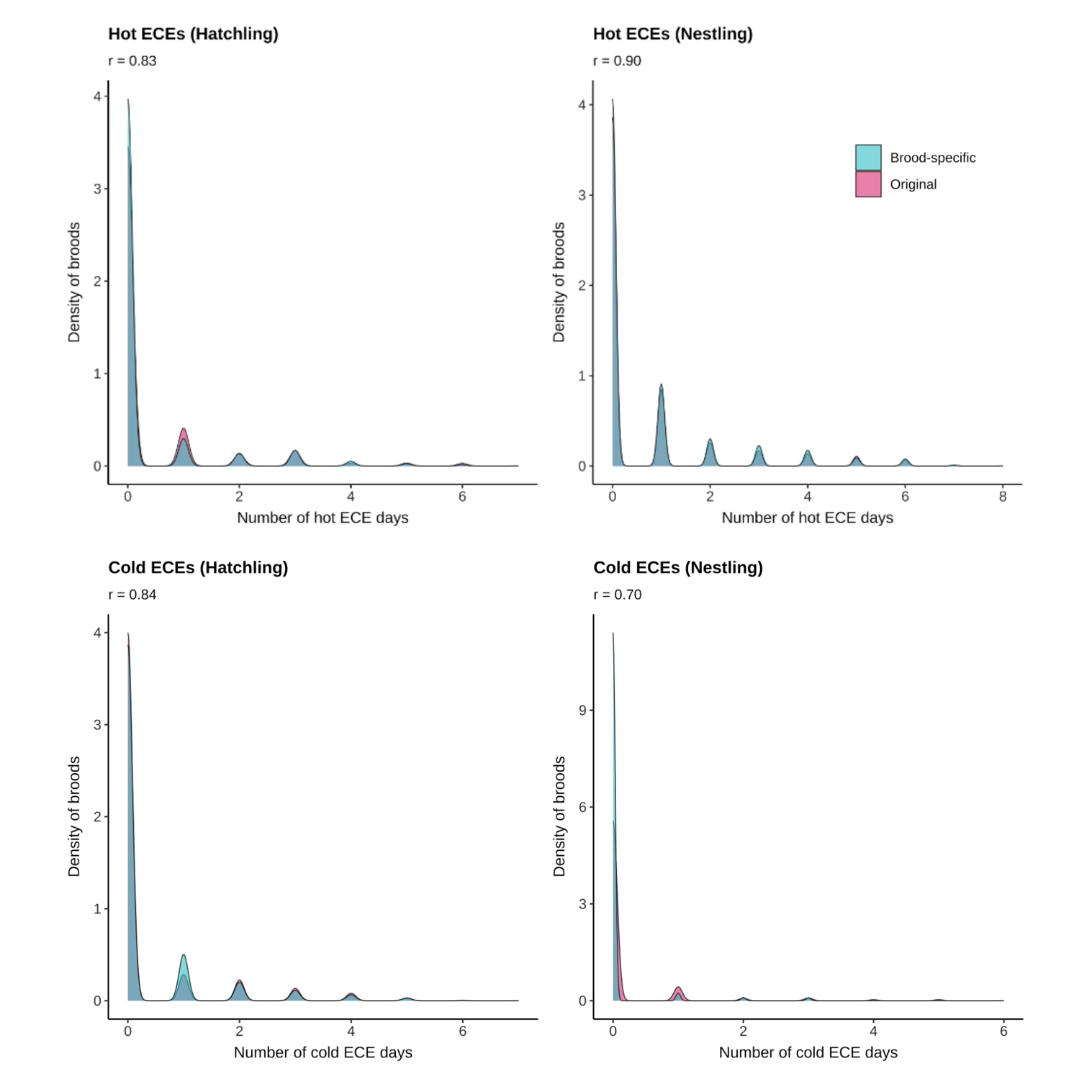


**Figure S2. Comparison of ECE frequency estimates under the original (monthly baseline) and brood-specific window methods.** Each plot shows distributions of the number of extreme temperature events (ECEs) per brood under the original monthly-baseline method (pink; “Original”) and the brood-specific ±15‑day window method (blue; “Brood-specific”). Plots show (A) hot ECEs during the hatchling stage, (B) hot ECEs during the nestling stage, (C) cold ECEs during the hatchling stage and (D) cold ECEs during the nestling stage. Reported Pearson correlation coefficients (r) indicate the strength of agreement in brood-level ECE counts between methods for each stage and ECE type.
